# Engineered dual affinity protein fragments to bind collagen and capture growth factors

**DOI:** 10.1016/j.mtbio.2023.100641

**Published:** 2023-04-22

**Authors:** Stylianos O. Sarrigiannidis, Oana Dobre, Alexandre Rodrigo Navarro, Matthew J. Dalby, Cristina Gonzalez-Garcia, Manuel Salmeron-Sanchez

**Affiliations:** Centre for the Cellular Microenvironment, Mazumdar-Shaw Advanced Research Centre, University of Glasgow, Glasgow, G116EW, UK

**Keywords:** Collagen, Bone regeneration, Recombinant protein fragment, Fibronectin, Biomaterials, Human mesenchymal stem cells, Bacteria

## Abstract

Collagen type I lacks affinity for growth factors (GFs) and yet it is clinically used to deliver bone morphogenic protein 2 (BMP-2), a potent osteogenic growth factor. To mitigate this lack of affinity, supra-physiological concentrations of BMP-2 are loaded in collagen sponges leading to uncontrolled BMP-2 leakage out of the material. This has led to important adverse side effects such as carcinogenesis. Here, we design recombinant dual affinity protein fragments, produced in *E. Coli*, which contain two regions, one that spontaneously binds to collagen and a second one that binds BMP-2. By adding the fragment to collagen sponges, BMP-2 is sequestered enabling solid phase presentation of BMP-2. We demonstrate osteogenesis *in vivo* with ultra-low doses of BMP-2. Our protein technology enhances the biological activity of collagen without using complex chemistries or changing the manufacturing of the base material and so opens a pathway to clinical translation.

## Graphical abstract

A protein fragment was engineered with the ability to bind both bone regenerating growth factors (i.e. rhBMP-2) and collagen. The protein fragment was expressed in bacteria and increased rhBMP-2 binding to collagen sponges compared to currently used clinical systems. The proposed technology showed excellent osteogenic potential *in vitro* and *in vivo* in critical-sized bone defect.

**Figure:**
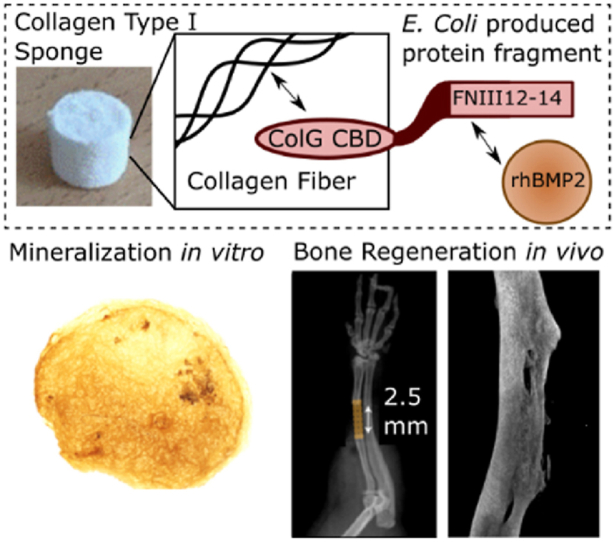

## 1 Introduction

Collagen scaffolds are widely used in tissue engineering. For bone tissue regeneration, a product called InFUSE bone graft (Medtronic) is a sector-leading material that is used as an alternative to autograft [1]. InFUSE is composed of an absorbable bovine collagen sponge (ACS) and recombinant human bone morphogenic protein 2 (rhBMP-2), a growth factor (GF) that has been shown to promote osteogenesis both *in vitro* and *in vivo* [2]*.* The system has received approval by the U.S. Food and Drug Administration (FDA) for bone regeneration applications and has since been used extensively for non-union tibial repairs, spinal fusion and maxillofacial reconstruction [2].

Despite successful implementation and outstanding results, InFUSE has nevertheless been linked to serious safety concerns. Patients treated with InFUSE bone graft have exhibited ectopic bone formation, tumor growth and severe inflammation among other severe side-effects [[3], [4], [5]]. InFUSE contains very high amounts of the GF (1.5 ​mg ​mL^−1^) which are far higher than the amounts observed physiologically during osteogenesis [3,4]. The reason for this is that collagen, in contrast to other extracellular matrix proteins such as laminin and fibronectin, which contain promiscuous GF binding sites, does not have a high affinity for BMP-2 which is unbound when loaded into the ACS and quickly leaks into the surrounding environment [6]. This means that high concentrations of the GF are required to achieve a therapeutic effect since a low dose would diffuse out of the collagen scaffold before having an effect, leading to the systemic side-effects described above.

Although the use of rhBMP-2 has been associated with health concerns, its potential for osseous tissue regeneration is undeniable and accelerating research into biomaterials which can control the release and reduce the amount of rhBMP-2 used is, therefore, important. Chen et al. [7] developed collagen binding BMP-2 by expressing the GF recombinantly linked to TKKLRT (a collagen binding sequence). The modified GF was loaded onto a demineralized bone scaffold and increased osteogenesis was observed in a subcutaneous rat and a rabbit mandible defect model. Instead of modifying GFs, Parmar et al. [8] developed a recombinant collagen with heparin-binding sequences, that bind GFs promiscuously, and which were shown to be capable of binding BMP-2, TGF-β and bFGF. These systems although effective, require extensive modification of the base material or GF which makes approval challenging. Modifying GFs is also a strategy that needs to be adapted on a case-by-case basis for tissue engineering applications, meaning that each GF required needs to be re-engineered to attach to the substrate of interest. Ideally a more flexible approach is required. Most recently Briquez et al. [6] developed a system where a bridge protein acts as the mediator between an ACS and rhBMP-2. The variable regions of an anti-collagen antibody were taken and linked to the heparin binding domain of laminin and expressed recombinantly in mammalian HEK 293-F cells. The ACS was treated with a solution containing the recombinant protein fragment before loading it with rhBMP-2. This system showed great promise in both a calvarial and spinal fusion mouse model. However, the need to use mammalian cells to produce the protein fragments makes the proposed solution overly expensive and thus impractical for large scale applications. Protein fragment expression utilizing an *E. Coli* bacteria-based system is an innovative proposition to alleviate, if successfully implemented, the issues of mammalian cell culture expression systems around cost-effective and scalability.

In this study, we developed a cost-effective, flexible and translatable approach to allow ACSs to bind and control the release of rhBMP-2 more effectively than the current systems. For this purpose, we took advantage of the ability of fibronectin to bind and stereoscopically present rhBMP-2 which occurs at fibronectin's highly promiscuous heparin-binding FNIII(12–14) domain which has been characterized extensively by multiple research groups [[9], [10], [11], [12]]. To achieve this, FNIII(12–14) was linked to various collagen binding domains (CBDs) from bacterial collagenases and placental GF to create three protein fragments which were recombinantly expressed in *E. Coli* bacteria. The CBD of fibronectin was considered but ultimately not trialed extensively due to low expression yields as a result of high cysteine content which *E. Coli* do not express well. The recombinant fragment was used to treat bovine ACSs, similar to the ones currently used in clinical practice (e.g. InFUSE), in an attempt to limit the amount of rhBMP-2 necessary to produce osteogenesis *in vitro* and *in vivo.*

We developed a protein fragment that can function as a linker between an ACS and rhBMP-2 which reduces the amount of BMP-2 necessary for osteogenesis and can be produced efficiently and in a cost-effective manner. We found that ACSs functionalized with a protein fragment combining the collagen binding domain of collagenase G linked with the FNIII(12–14) domain absorb and retain rhBMP-2 more effectively than untreated ACSs. The protein fragment treated ACSs promote *in vitro* osteogenesis using rhBMP-2 at a concentration of only 2 ​μg ​mL^−1^ while untreated collagen scaffolds did not showcase the same result. *In vivο,* protein fragment-functionalized ACSs loaded with a dose as low as 2.5 ​μg/mL showed new bone formation in a mouse radial bone critical size defect model. When higher concentrations of BMP-2 (75 ​μg ​mL^−1^, still very low compared to 1.5 ​mg ​mL^−1^ for InFUSE) were used the defect gap was completely bridged. Since the GF binding domain of fibronectin can bind multiple GFs, the system can be expanded for more tissue engineering applications in the future.

## 2 Results

Note: Unless otherwise specified, an ACS was used as a substrate in every experimental condition discussed.

### 2.1 Protein fragments can be produced with affinity to collagen

**Fig. 1:**
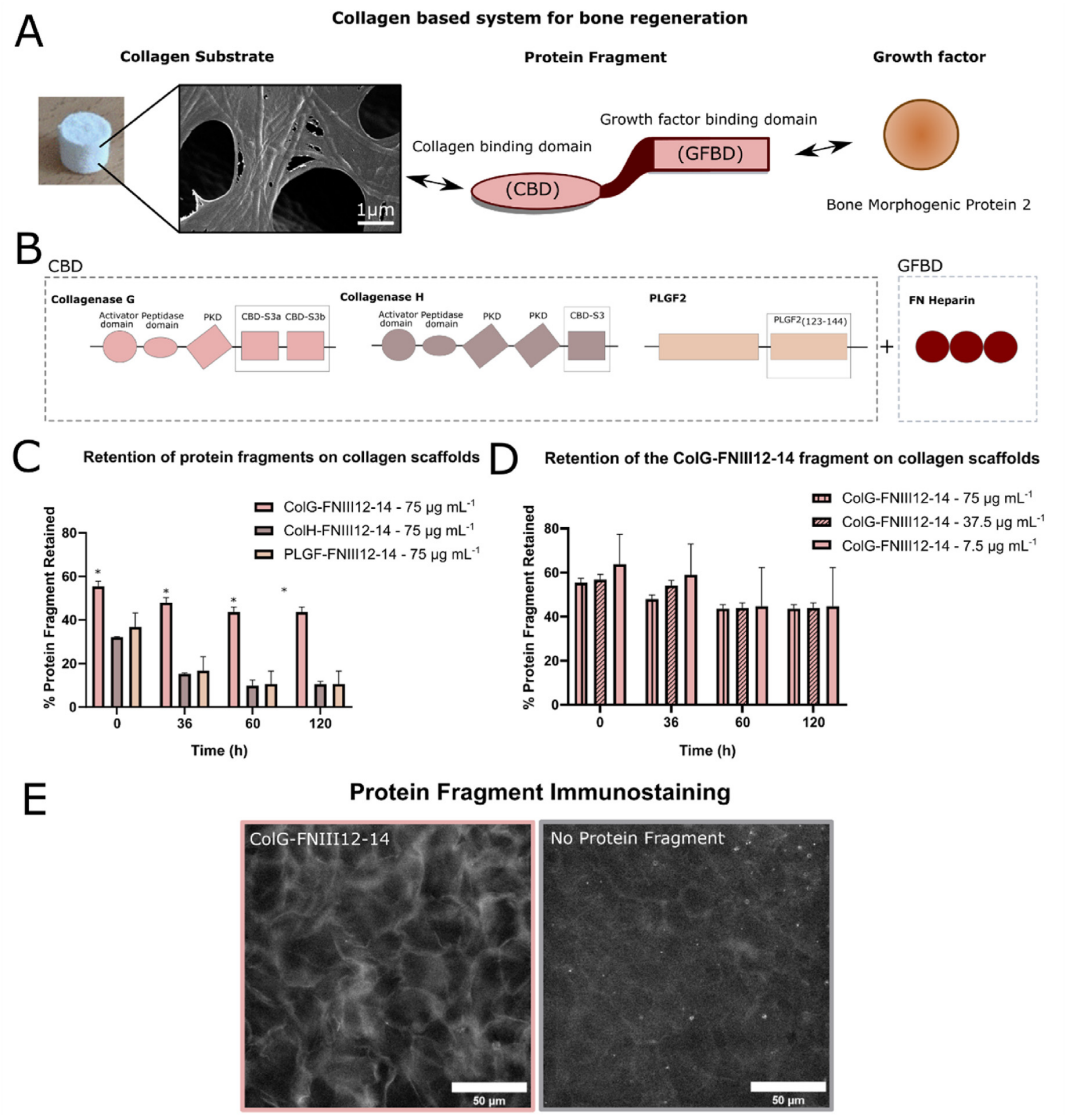
A) Outlines the proposed system of using a protein fragment to create a link between a collagen substrate and GFs such as rhBMP-2. To achieve this a collagen-binding domain (CBD) is linked to a growth factor binding domain (GFBD) (Scale bar 1 ​μm). B) The CBDs that were explored were from collagenase G (S3a-S3b), collagenase H (S3) and PLGF (PLGF(123–144)). The GFBD explored was the FNIII(12–14) domain as it is easy to produce in bacteria and has been extensively studied. C) Shows how the protein fragments with varying CBDs (namely the collagenase G-FNIII12-14 (ColG-FNIII12-14), collagenase H–FNIII12-14 (ColH-FNIII12-14), PLGF-FNIII12-14) performed when collagen substrates were treated with the respective protein fragment. The protein fragment with collagenase G as the CBD was retained the most by the collagen substrate and was chosen for further evaluation. D) Different concentrations of the ColG-FNIII12-14protein fragment were retained similarly in terms of percentage (%) by the collagen scaffold but the mass amount of the protein fragment retained was higher the higher the concentration of ColG-FNIII12-14 initially loaded. E) An immunostaining image of the collagen scaffold with and without the protein fragment can be seen which clearly shows how the protein fragment decorates the collagen scaffold (Scale bar 50 ​μm). Statistical significance (∗p ​< ​0.05).

A protein fragment, to be recombinantly produced in *E. Coli* bacteria, was designed that can function as a linker between rhBMP-2 and an ACS (Fig. 1 A), since collagen lacks the ability to bind and retain BMP-2 efficiently. The growth factor binding domain (GFBD) used was based on the heparin II binding region of fibronectin (FNIII12-14) which has been extensively characterized and shown to promiscuously bind multiple GFs, including BMP-2 [9,11,12].

For the collagen binding domains (CBD), regions from clostridium histolyticum collagenase G (S3a ​+ ​S3b) and collagenase H (S3) [13,14] as well as placental growth factor's PLGF2_123-144_ region [15] were tested as they have shown promising collagen binding capabilities (Fig. 1 B) [13,14]. Therefore, three protein fragments were designed with the same GFBD but varying CBD linked with a glycine-serine linker to separate each domain. The protein fragments were expressed in *E. Coli* bacteria using a pet100 vector and isolated using a his-tag binding column.

The ability of these protein fragments to bind collagen was assessed through a micro-BCA. ACSs (150 ​μL in volume) were coated with 75 ​μg of each protein fragment and the release of the protein fragments from the scaffold was monitored over time as a percentage of the initial amount of protein fragment absorbed (Fig. 1C). It was determined that ColG-FNIII12-14 was retained most effectively by the ACSs with 43% of the protein fragment absorbed remaining bound after 120 ​h. In contrast, only around 10% of the PLGF-FNIII12-14 and ColH-FNIII12-14 protein fragments absorbed were bound to the ACS in the same timeframe.

Collagenase G has higher affinity (10×) for collagen protein fragments and insoluble collagen compared to collagenase H [14], likely due to the increased length of the collagenase G CBD compared to the CBD in collagenase H, and our results are consistent with that finding. It has been suggested that 1 ​mg of collagen type 1 can bind 108 ​pmol of collagenase H [16]. The amount of ColH- FNIII12-14 protein fragment remaining on ACSs in our study was calculated to be 90 ​pmol per 1 ​mg of collagen after 120 ​h which is similar to the literature value reported [16]. Although the maximum collagenase G binding capacity of collagen type 1 is not known, we found that approx. 325 ​pmol of ColG-FNIII12-14 protein fragment was remaining on the collagen scaffolds after 120 ​h which is 3.6× higher than for ColH- FNIII12-14. PLGF has been shown to have a high affinity for ECM proteins [15] but our results suggest that binding is less effective in ACSs. However, both the origin and the solubility of the collagen used in the study [15] is not known which makes a direct comparison to our results not possible.

Varying amounts of ColG-FNIII12-14 protein fragment were used to treat the ACSs in order to explore the concentration dependent retention of the protein fragment when absorbed onto collagen. No statistically significant difference, in terms of percentage retention, was observed between 75 ​μg (8 μΜ), 37.5 ​μg (4 ​μM) and 15 ​μg (1.6 ​μM) of protein fragment loaded into the ACSs (150 ​μL in volume) after 120 ​h (Fig. 1 D). However, the total mass of ColG-FNIII12-14 protein fragment left in the ACS was 32 ​μg (3.5 μΜ), 16 ​μg (1.7 μΜ) and 6.5 ​μg (0.69 μΜ) respectively (Fig. 1 D), suggesting that the higher the protein fragment concentration used to treat the ACSs, the higher the amount of protein fragment retained. This indicates that the ACS was not saturated with protein fragment. However, higher concentrations of the ColG-FNIII12-14 protein fragment were redundant considering that the molar concentration of GF (rhBMP-2) to be used at a later stage in conjunction with the protein fragment was substantially lower than the molar concentration of the protein fragment. Even when 37.5 ​μg (4 μΜ) of ColG- FNIII12-14 was used, the highest concentration of rhBMP-2 loaded into our system was 77 ​nM (*in* vitro) and 2.88 ​μM (*in vivo*). To further showcase ColG-FNIII12-14 and collagen interaction, his-tag immunostaining (all protein fragments are expressed with a his-tag) was also performed on both ColG-FNIII12-14 treated and un-treated ACSs. The ColG- FNIII12-14 protein fragment is clearly present in the ACS and outlines the porous structure of the scaffold since the fragment accumulates on the collagen fibres forming the pores. Similar features cannot be seen in the un-treated ACS treatment (Fig. 1 E).

### 2.2 Protein fragments treated collagen scaffolds support cell culture and attachment

**Fig. 2:**
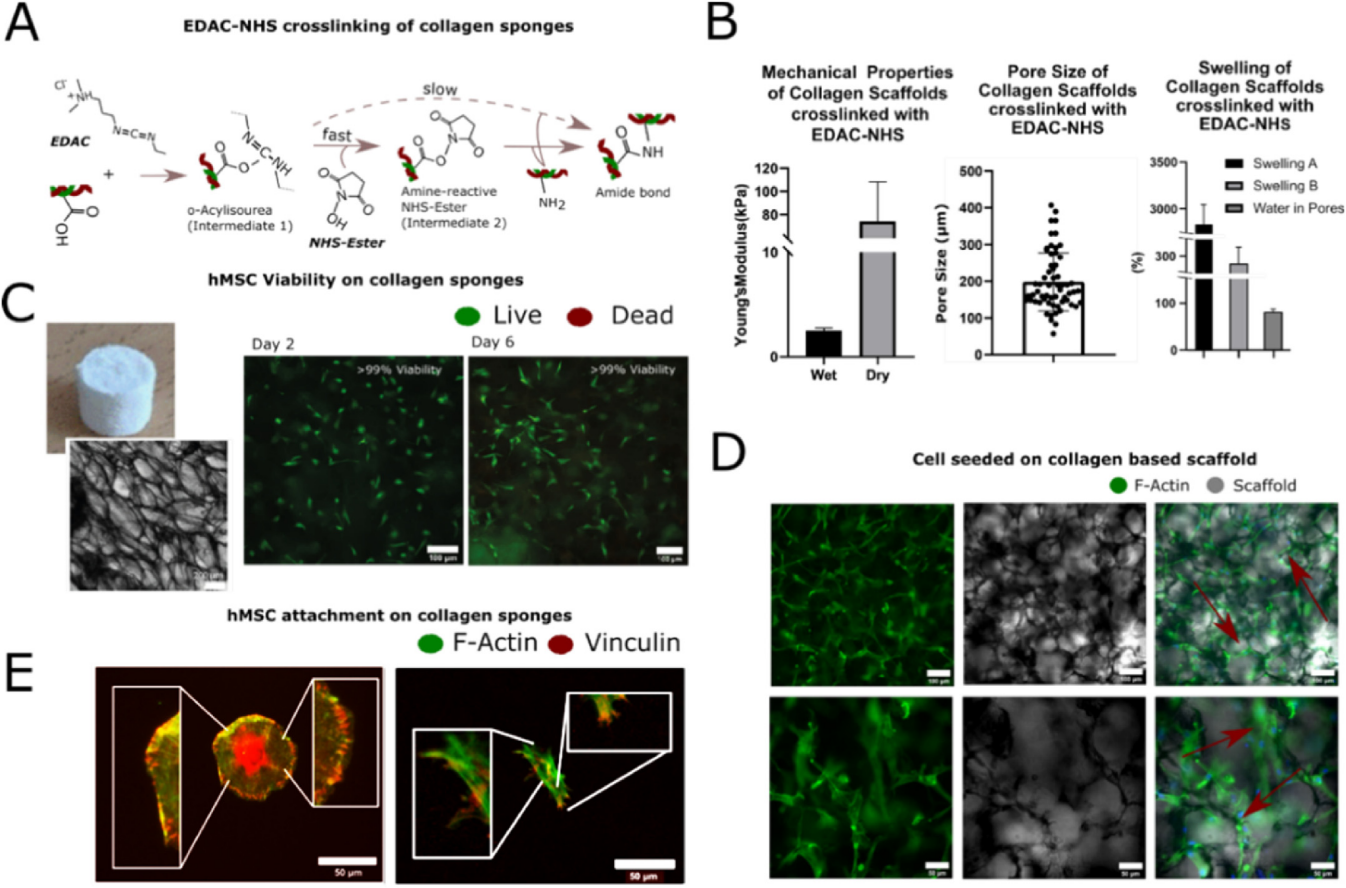
A) Shows the chemistry used to crosslink the collagen scaffolds before they are treated with the protein fragment. B) Shows the mechanical properties, pore size and swelling characteristics of the collagen scaffolds. Scaffolds were 2.5±0.47 ​kPa in wet conditions and 74.25±30 ​kPa in dry conditions, the pore size ranged from 57 ​μm to 407 ​μm with a mean of 198 ​μm, total scaffold swelling (swelling A) was 2743±300 (%), fiber swelling (swelling B) 286±30 (%) and the water in pores was measured to be 85±4 (%). The mechanical properties are consistent with collagen scaffolds in literature, the pore size is sufficient for osteogenesis, and the swelling suggests great scaffold wettability and water retention important for cell culture. C) The viability of hMSC on collagen scaffolds was found to be greater than 99% (Scale bar 100 ​μm) even after the addition of the protein fragment and D) cells were spindle-like and spread along the pores of the scaffolds (Scale bar top: 100 ​μm bottom: 50 ​μm). E) Vinculin staining revealed that hMSCs formed focal adhesions when interacting with collagen (right image) similar to the ones seen on glass (left image) suggesting integrin-mediated interaction with the substrate despite the addition of the protein fragment (Scale bar 50 ​μm).

Before functionalizing the ACSs with ColG-FNIII12-14, or other protein fragments, they were crosslinked with EDAC-NHS at a molar concentration of 5:2:1 (EDAC:NHS:COOH) which is a standard crosslinking protocol used for collagen scaffolds (Fig. 2 A). The scaffolds were also analyzed in terms of their mechanical properties, pore size and swelling properties (Fig. 2 B). Collagen scaffolds had a Young's modulus of 74.25±30 ​kPa in dry conditions and 2.5±0.47 ​kPa in wet conditions. The mechanical properties are consistent with literature values and similar to other collagen scaffolds used for bone regeneration [17,18]. Scaffolds showed pores of different sizes which were interconnected and randomly distributed. The pore size ranged from 57 to 407 ​μm with an average of 198 ​μm, which is a pore size range allowing cell infiltration and cell metabolic activity for bone tissue regeneration [19,20]. The collagen scaffolds also showed desirable swelling properties. Total scaffold swelling was 2743±300% (Swelling A), fiber swelling was calculated at 286±30% (Swelling B) and the water in pores was measured to be 85±4%. These findings are consistent with what has been seen in the literature and suggests that collagen scaffolds have good wettability and water retention properties which is important for cell viability and growth [18,21].

Cell viability on collagen scaffolds treated with the ColG-FNIII12-14 protein fragment was measured after culturing hMSCs on the collagen substrate at a seeding density of 330,000 ​cells per mL for up to 6 days. At both day 2 and day 6 of the experiment, hMSC viability was found to be higher than 99% (Fig. 2 A). Morphologically, cells showed extensive spreading with clearly visible f-actin stress fibers (Fig. 2 D).

HMSCs could be seen aligning with the porous morphology (Fig. 2D) of the collagen substrate, this has also been observed by Zhang et al. [22]. Cell attachment to the collagen scaffold treated with ColG-FNIII12-14 was also examined by seeding hMSCs at a density of 67,000 ​cells/mL and staining for actin and vinculin to investigate focal adhesion formation. Focal adhesions are structures composed of multiple proteins including focal adhesion kinase (FAK), paxillin and vinculin which allow cell mechano-sensing; vital for correct cell function and viability [23]. Vinculin clusters overlapping with actin filaments can be seen in both the glass as well as the ColG- FNIII12-14 treated collagen scaffolds (Fig. 2 E), suggesting the formation of focal adhesion and integrin-mediated hMSCs collagen interaction. Overall, hMSCs viability, morphology and focal adhesion formation were found to be in agreement with literature [24,25].

### 2.3 Protein fragment treated ACSs efficiently bind rhBMP-2

After showing that our collagen-protein fragment system supports cell culture, we explored the ability of the ColG-FNIII12-14 protein fragment to bind rhBMP-2, allowing ACSs to retain rhBMP-2 effectively. Carrier free rhBMP-2 was tagged with a fluorophore (Dylight-488) and absorbed onto ACSs with and without ColG-FNIII12-14 for 1 ​h. An aliquot was taken pre- and post-absorption and the fluorescence of the aliquots was measured at 490 ​nm. The values were converted to mass amounts using a standard curve of known 488-tagged rhBMP-2 concentrations and the percentage absorption was calculated according to the following equation:

$$Percentage\;absorption\;\left(\%\right)=\frac{PreAbsorption-PostAbsorption}{PreAbsorption}x100$$

**Fig. 3:**
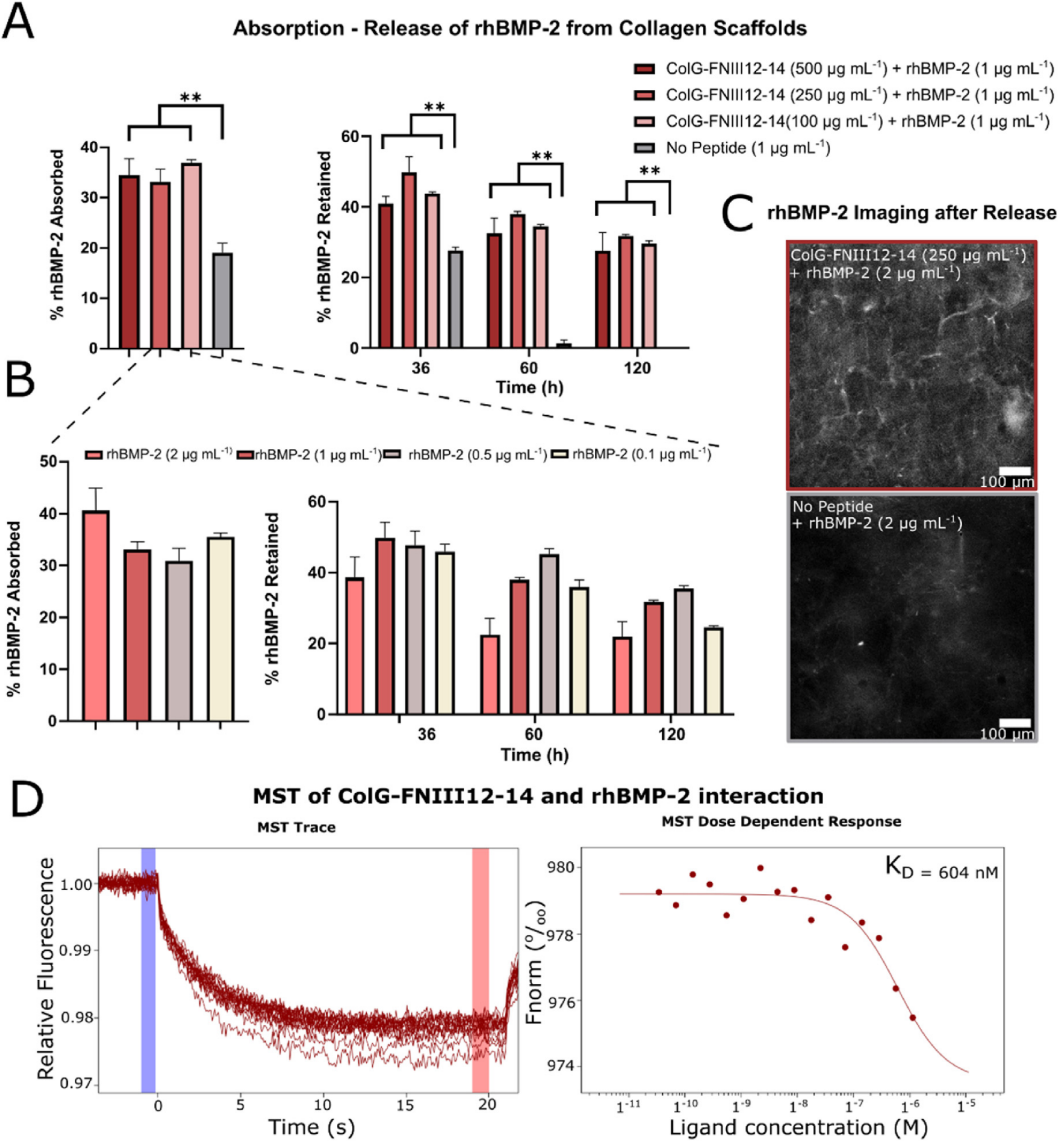
A) Shows the % rhBMP-2 (1 ​μg ​mL^−1^) absorbed on collagen scaffolds treated with different concentrations of ColG-FNIII12-14 protein fragment and compared to rhBMP-2 absorption onto non-protein fragment treated collagen scaffolds. The release of the rhBMP-2 absorbed onto the scaffolds was also monitored for a 120 ​h period. The data suggests that there is significantly higher rhBMP-2 absorption and retention in the conditions where the collagen scaffolds were treated with protein fragments compared to the no protein fragment condition. There was no significant difference between using different amounts of protein fragment. B) Keeping the amount of protein fragment the same (250 ​μg ​mL^−1^) and varying the amount of rhBMP-2 from 0.5 to 2 ​μg ​mL^−1^ also did not affect the percentage absorption and release but the absolute amount in μg of protein fragment absorbed and retained did increase with increasing concentrations. C) An image visualising rhBMP-2 was taken after the conclusion of the absorption-release experiments and the protein fragment treated collagen substrate was compared to the no treatment condition. It can be clearly seen that the substrate with the protein fragment retained more BMP-2 compared to the collagen scaffold without protein fragment (Scale bar 100 ​μm). D) The interaction strength between the protein fragment and rhBMP-2 was also assessed using MST. The Kd of the interaction was found to be around 604 ​nM which suggest a medium high affinity for rhBMP-2 and the protein fragment. Statistical significance (∗∗p ​< ​0.01).

We observed that 18% of rhBMP-2 initially binds to collagen (Fig. 3 A). However, ColG-FNIII12-14 treated collagen scaffolds absorbed more rhBMP-2 comparatively. Varying the amount of ColG-FNIII12-14 protein fragment used to treat collagen did not influence the percentage of rhBMP-2 absorbed (Fig. 3 A) with all conditions absorbing 30–32% of rhBMP-2. However, as the molarity of rhBMP-2 used in this assay was 38 ​nM while the amount of protein fragment remaining in the collagen system even at the lowest concentration was 690 ​nM which provides each rhBMP-2 molecule 18 possible binding sites. The release of rhBMP-2 absorbed on ACSs was also explored by submerging the ACSs in PBS for up to 120 ​h and reading the fluorescence of the supernatant at 36, 60 and 120 ​h (after 120 ​h no further considerable release was observed as seen in Supplementary Fig. 2). Each time a measurement was taken, PBS was refreshed. The amount of rhBMP-2 retained in the scaffold was calculated as a percentage of the rhBMP-2 absorbed, as had been calculated earlier. Similar trends to the absorption experiment were seen with ColG-FNIII12-14 treated collagen scaffolds retaining significantly more GF after 120 ​h (33% of the rhBMP-2 absorbed) compared to the plain collagen substrate (0% of the rhBMP-2 absorbed).

For further experimentation, 37.5 ​μg (4 ​μM) of the ColG-FNIII12-14 protein fragment was used to treat collagen scaffolds to ensure the system was saturated and enough GF binding sites were available in case higher concentrations of rhBMP-2 were required. The absorption and release of rhBMP-2 was monitored while keeping the ColG-FNIII12-14 concentration constant and varying the amount of rhBMP-2 (0.1, 0.5, 1 and 2 ​μg ​mL^−1^ – 3.85, 19.25, 38.7 and 77 ​nM) (Fig. 3 B). GF concentrations used in hydrogels for *in vitro* applications in the past range from 5 to 100 ​μg ​mL^−1^ [[26], [27], [28]]. The percentage amount absorbed and retained in the protein fragment treated collagen did not vary significantly between conditions showing 30–41% absorption and 23–35% retention (of the amount absorbed) after 120 ​h (Fig. 3 B). Trujillo et al. [11] similarly observed that the percentage release of rhVEGF from fibronectin was similar for different concentration of the GF. Nevertheless, the rhBMP-2 remaining in the system increased when higher concentrations of GF were absorbed on the protein fragment treated ACSs (0.009, 0.055, 0.105 and 0.192 ​μg ​mL^−1^ (0.4, 2.1, 4 and 7.4 ​nM) of rhBMP-2 was left in the ACSs after treating the substrates with 0.1, 0.5, 1 and 2 ​μg ​mL^−1^ (3.85, 19.25, 38.7 and 77 ​nM) of rhBMP-2 respectively) (Fig. 3 B).

Since the rhBMP-2 used was tagged with a fluorophore, images were taken with a fluorescence microscope after the conclusion of the absorption-release experiment (120 ​h). The ColG-FNIII12-14 treated ACSs showed increased fluorescence intensity compared to the non-treated protein fragment treated ACS, suggesting further that the ColG-FNIII12-14 protein fragment aids in rhBMP-2 retention (Fig. 3C).

Lastly, microscale thermophoresis (MST) was carried out to calculate the Kd value of the interaction between the ColG-FNIII12-14 protein fragment and fluorescently tagged rhBMP-2. The molar concentration of rhBMP-2 was kept constant at 20 ​nM while the molar concentration of ColG-FNIII12-14 was increased up to 1 μΜ. A Kd value of 604 ​nM was calculated which signifies medium high affinity for the protein fragment and rhBMP-2 (Fig. 3 D). This value is lower compared to the one quoted in literature for BMP-2 fibronectin interactions (Kd ​= ​5.26±1.25 ​nM) [29]. However, using full-length fibronectin is costly and needs to undergo extensive processing to unfold as it is naturally a dimer adopting a globular conformation on surfaces which shields its GFBD. Surface modifications with polymers such as poly-ethyl glycol or fibronectin denaturation are required to expose the hidden GFBD and enable GF-fibronectin interaction [11,12]. Using a recombinant fragment of the protein to achieve a similar result is a more cost-effective and efficient approach.

### 2.4 Protein fragment treated collagen scaffolds showed increased osteogenesis *in vitro*

We have shown that treating an ACSs with ColG-FNIII12-14 – a recombinant, collagen and GF binding protein fragment – allows the ACS to sequester and retain significantly more rhBMP-2 compared to the non-treated ACS as shown in 2.3. Increased rhBMP-2 retention has been associated with improved osteogenesis in the past [11,26]. To explore the potential of the proposed system for bone regeneration *in vitro* and *in vivo* experiments were carried out.

**Fig. 4:**
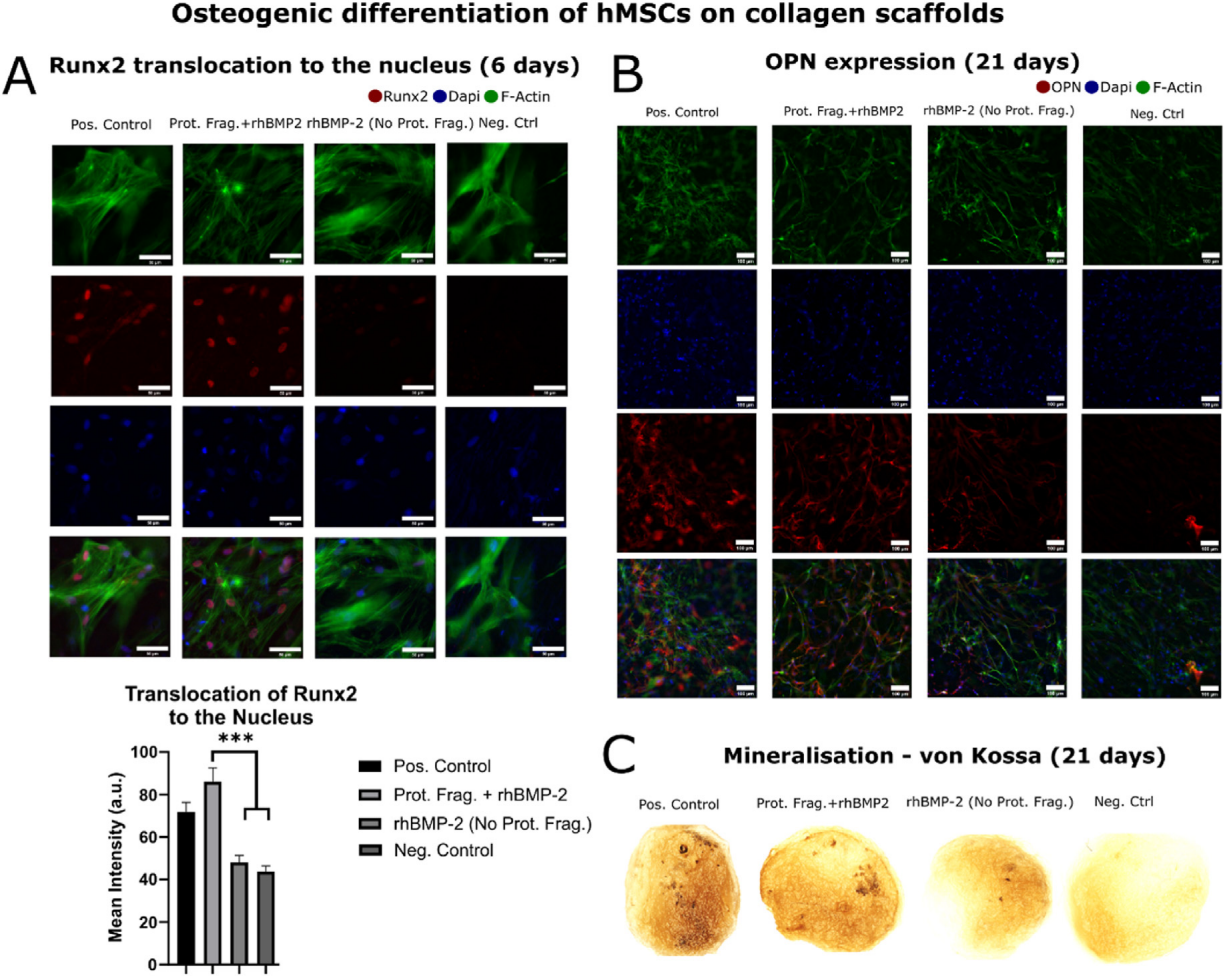
Shows hMSC osteogenic differentiation on a collagen scaffold (neg. control), a collagen scaffold loaded with 2 ​μg ​mL^−1^ of rhBMP-2 (rhBMP-2 (no protein fragment)), a protein fragment treated collagen scaffold loaded with 2 ​μg ​mL^−1^ of rhBMP-2 (protein fragment ​+ ​rhBMP-2) and collagen scaffold cultured in osteogenic media (pos. control) A) Runx2 translocation to the nucleus was investigated after 6 days using immunostaining and measuring the mean intensity of Runx2 staining within the nucleus. The protein fragment treated collagen scaffolds with rhBMP-2 showed significantly higher Runx2 translocation to the nucleus compared to the neg. control and the collagen scaffold with rhBMP-2 but no protein fragment. The protein fragment ​+ ​rhBMP-2 condition was not statistically different to the osteogenic media condition which also showed increased translocation to the nucleus (Scale bar 50 ​μm). B) The same trend was observed when culturing cells for 21 days and staining for osteopontin and C) mineral deposition. The protein fragment ​+ ​rhBMP-2 condition performed most similar to the osteogenic media but better than both the neg. control and the rhBMP-2 (no protein fragment) conditions (Scale bar 100 ​μm). Statistical significance (∗∗∗p ​< ​0.001).

We investigated the ability of our ColG-FNIII12-14 treated collagen scaffolds to promote hMSC osteogenic differentiation when loaded with rhBMP-2 in comparison to non-treated collagen scaffolds. Runx-2 is an important transcription factor which translocates to the nucleus when BMP-2 mediated osteogenic differentiation of hMSCs is initiated [30]. This occurs in the early stages of osteogenesis. Fig. 4 A shows immunofluorescence images of hMSCs cultured for 6 days on ACSs with osteogenic media (positive control), treated with ColG-FNIII12-14 (250 ​μg ​mL^−1^) ​+ ​rhBMP-2 (2 ​μg ​mL^−1^ – 77 ​nM), non-treated ​+ ​rhBMP-2 (2 ​μg/mL – 77 ​nM) and with no treatment (negative control) with basal media. The ColG-FNIII12-14 treated ACS with rhBMP-2 showed translocation of Runx2 (in red) to the nucleus, similarly to osteogenic media (positive control). Runx2 intensity within the boundaries of the nucleus was also quantified using ImageJ and followed a similar trend (Fig. 4 A). The mean intensity (normalized to the area of the nucleus) of the ColG- FNIII12-14 protein fragment ​+ ​rhBMP-2 condition, and osteogenic media (positive control) was significantly higher compared to the no-protein fragment treated ACS ​+ ​rhBMP-2 and plain ACS (negative control) conditions. The no-protein fragment ​+ ​rhBMP-2 condition did not showcase statistically significant upregulation of Runx2 compared to the negative control because, as previously discussed, rhBMP-2 escapes the ACS substrate in the absence of the ColG-FNIII12-14 protein fragment, not allowing sufficient time for the growth factor to have a significant effect on hMSC osteogenesis. Conversely, the ColG-FNIII12-14 protein fragment ​+ ​rhBMP-2 condition shows statististically significant Runx2 upregulation since growth factor release is delayed.

Osteopontin (OPN), a late osteogenic marker [30], expression was also investigated after culturing hMSCs for 21 days [31]. Immunofluorescence images were taken and clearly show that OPN (in red) is upregulated in both the positive control (osteogenic media) and the ColG-FNIII12-14+ rhBMP-2 (2 ​μg ​mL^−1^ – 77 ​nM) condition (Fig. 4 B). Less OPN was expressed in collagen scaffolds without the protein fragment, despite the addition of 2 ​μg ​mL^−1^ – 77 ​nM rhBMP-2 and almost no OPN could be clearly identified in the plain ACS (negative control) (Fig. 4 B). This confirms the findings observed when exploring early osteogenic markers such as Runx2. Mineralization staining (von Kossa) after 21 days [31] confirms this, also showing that the ColG-FNIII12-14 ​+ ​rhBMP-2 condition performed better than the rhBMP-2 (no protein fragment) samples and showed more mineral nodules, similar to positive control (osteogenic media) (Fig. 4C). As expected hMSCs cultured in basal media on a plain ACS (negative control) did not show any collagen mineralization (Fig. 4C).

### 2.5 Protein fragment-treated collagen scaffolds showed increased osteogenesis *in vivo*

After demonstrating osteogenesis *in vitro,* ACSs (Supplementary Fig. 3) treated with ColG-FNIII12-14 and rhBMP-2 were implanted in a (non-healing) critical size defect mouse model (Supplementary Fig. 5) [11], to evaluate their potential to promote bone regeneration *in vivo*.

**Fig. 5:**
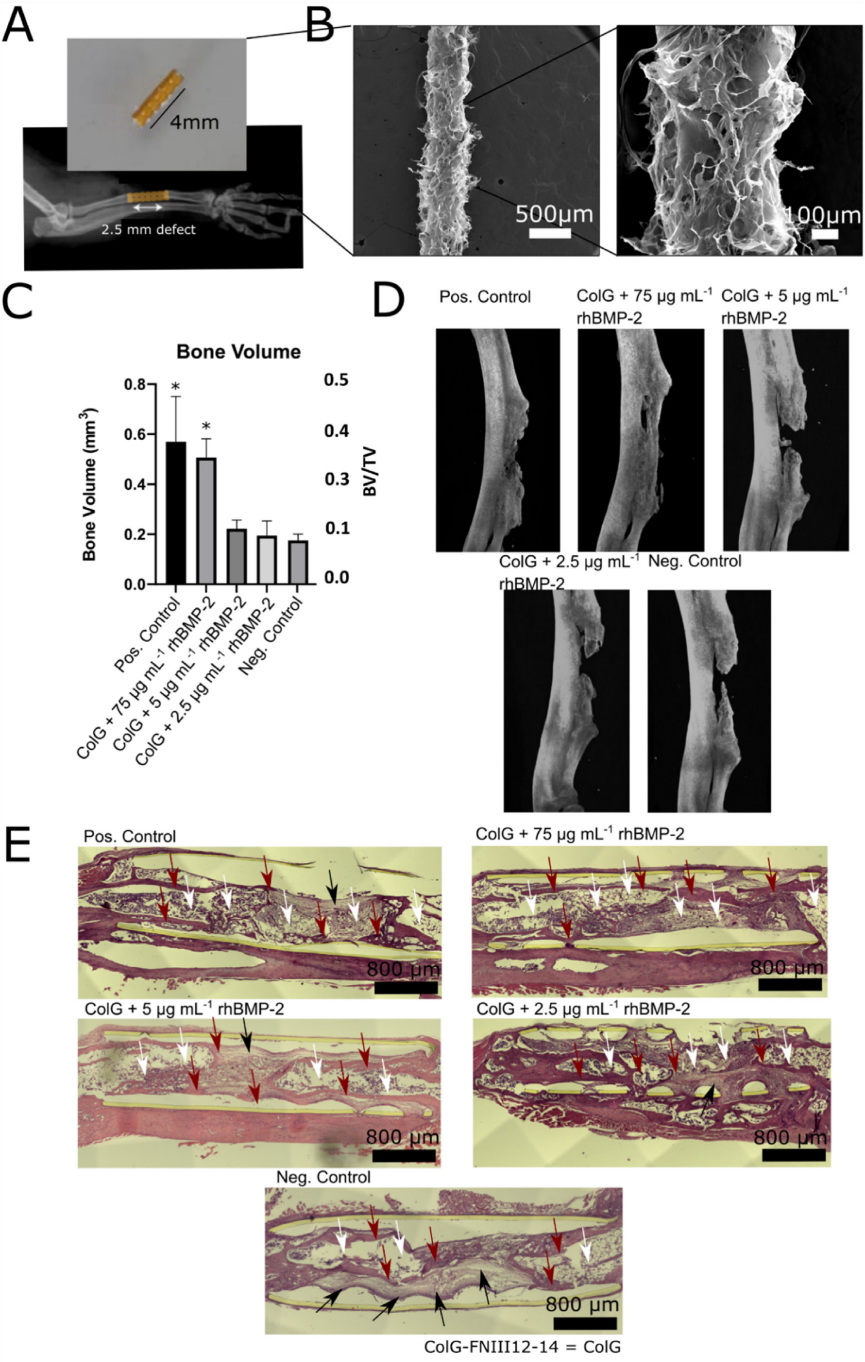
Protein fragment-functionalized ACSs promote new bone growth *in vivo.* A) Picture of the implant tube filled will a ACS and an X-ray image of the critical size (2.5 ​mm) murine radial defect model used. B) SEM images of the ACSs implanted. C) The new bone volume (NBV) and BV/TV (NBV/Total Volume) formation was quantified in the positive control (ACS with 75 ​μg ​mL^−1^ rhBMP-2 with no added ColG-FNIII12-14 protein fragment), 250 ​μg ​mL^−1^ ColG-FNIII12-14 ​+ ​75 ​μg ​mL^−1^ rhBMP-2, 250 ​μg ​mL^−1^ ColG-FNIII12-14 ​+ ​5 ​μg ​mL^−1^ rhBMP-2, 250 ​μg ​mL^−1^ ColG-FNIII12-14 ​+ ​2.5 ​μg ​mL^−1^ rhBMP-2, and plain ACS (negative control) condition (mean±SEM, n ​= ​6, p ​< ​0.05). D) 3D reconstruction of micro-computerized tomography (μCT) scans of the area where the implant was placed in the critical size murine radial defect. The samples closest representing the mean are depicted. E) Histological images of the same samples depicted in (D) showing hematoxylin and eosin staining. Black arrows point to fibrotic tissue, white arrows point to the formation of a bone marrow cavity/cancellous bone, while maroon arrows point to new cortical bone. Statistical significance (∗p ​< ​0.05).

A critical-size 2.5 ​mm defect, that does not heal on its own, was created in the right radial bone of the mouse. The ulna was left untouched to act as a stabilizing structure, avoiding the use of additional external fixation plates. 4 ​mm implant tubes were filled with 3 ​μL collagen slurry 1% w/v, freeze dried, crosslinked with EDAC-NHS and freeze-dried a second time to yield small ACSs (Fig. 5 and Supplementary Fig. 3).

The implant tubes containing the ACSs were sterilized with ethylene oxide and treated with the corresponding protein fragment and/or rhBMP-2 GF (ACS ​+ ​75 ​μg ​mL^−1^ rhBMP-2 (positive control), 250 ​μg ​mL^−1^ ColG-FNIII12-14 ​+ ​75 ​μg ​mL^−1^ rhBMP-2, 250 ​μg ​mL^−1^ ColG-FNIII12-14 ​+ ​5 ​μg ​mL^−1^ rhBMP-2, 250 ​μg ​mL^−1^ ColG-FNIII12-14 ​+ ​2.5 ​μg ​mL^−1^ rhBMP-2, neg. control - no ColG-FNIII12-14 ​+ ​no BMP-2).

rhBMP-2 adsorption on the ACS within the implant tube was confirmed by submerging the sponges in a solution of rhBMP-2 tagged with Dylight 488® for 1h. The sponges in the implant tubes were then rinsed with water, to remove the unbound rhBMP-2, and imaged using a fluorescent microscope. When looking at Supplementary Fig. 5, it can be clearly seen that the fluorescent intensity of the sponges treated with rhBMP-2 is higher compared to the non-treated ones, suggesting that the rhBMP-2 solution is absorbed by the sponges even when inside the implant tube.

The breakdown of the sponges inside the implant tube was then explored to ensure that they would degrade when implanted *in vivo* within the timeframe of the experiment. As the weight change of the scaffold was too low to record accurately, we opted to measure the change in sponge dimensions when incubated at 37 ​°C in PBS or collagenase type 1 ​at 10 U ​mL^−1^. The sponges did not hydrolytically degrade as fast as when collagenase was introduced. After 5 weeks approx. 50–60% of the sponge was left when incubated in PBS, while less than 10% was left when incubated with collagenase type 1. This is expected as the digestion of collagen with collagenase is a well-known metabolic pathway, while crosslinked collagen does not hydrolytically degrade as fast [32,33]. The degradation of the ACSs is important because non-degradable materials negatively impact new bone formation. Ideally, the degradation profile should match the formation of new bone or occur in a similar time frame. As seen in Supplementary Fig. 4, the degradation profile of the sponges inside the implant tubes took place within 5 weeks which is aligned to the timeline of the *in vivo* experiment (8 weeks).

Eight weeks after *in vivo* implantation, the mice forearm bone samples were explanted and analyzed (the results are summarized in Table 1) by micro-computerized tomography (μCT) to quantify new bone formation. Analysis of the bone defects showed that in the ACS +75 ​μg ​mL^−1^ rhBMP-2 (positive control) as well as the ColG-FNIII12-14 ​+ ​75 ​μg ​mL^−1^ (2.88 ​μM) rhBMP-2 condition showed total bridging of the gap in 3 out of 6 conditions. The quantification of the μCT scans also showed that there was no difference between the two conditions with both showing substantial new bone volume (NBV) formation and a higher BV/TV. Note that in the representative μCT reconstruction of the ACS +75 ​μg ​mL^−1^ rhBMP-2 (positive control) (Fig. 5C) the NBV appears less compared to the ColG-FNIII12-14 ​+ ​75 ​μg ​mL^−1^ (2.88 ​μM) as the gap is not fully bridged. Nevertheless, the NBV in both is similar due to denser bone in the proximal and distal ends of the implant in former condition compared to the latter. A complete bridging of the defect was not observed in any other condition. NBV was observed in all remaining conditions with the plain ACS (negative control) showing the least NBV. The sponges treated with ColG-FNIII12-14 ​+ ​either 2.5 or 5 ​μg ​mL^−1^ (96.25 or 192.5 ​nM) rhBMP-2 performed better than the plain ACS (negative control) albeit the NBV or BV/TV measured was not statistically significant (Fig. 5C). The trabecular bone pattern factor (Tb.pf) – a measure of bone connectivity and structural integrity i.e. bone quality (note: lower values indicate greater bone quality) – was calculated for all conditions. The lowest Tb.pf (0.010±0.002 ​mm^−1^) i.e. highest quality bone was observed for the ColG-FNIII12-14 ​+ ​75 ​μg ​mL^−1^ (2.88 ​μM) rhBMP-2 and was significantly lower than the Tb.pf for 75 ​μg ​mL^−1^ (2.88 ​μM) rhBMP-2 (positive control) (0.021±0.004 ​mm^−1^). The Tb.pf value for the positive control was in fact closest to the ColG-FNIII12-14 ​+ ​5 ​μg ​mL^−1^ (192.5 ​nM) rhBMP-2 condition (0.021±0.005 ​mm^−1^). This further suggests that treating an ACS with our fragment significantly improves new bone quality. No further significant differences were observed but the highest values of Tb.pf i.e. lowest bone quality were observed for the plain ACS (negative control) (0.039±0.018 ​mm^−1^) and the ACS treated with ColG-FNIII12-14 ​+ ​either 2.5 (96.25 ​nM) rhBMP-2 (0.058±0.039 ​mm^−1^) (Supplementary Fig. 7) as expected.

**Table 1:** Table summarizing *in vivo* results. Legend: , +, +(+), ++, ++(+), +++ (ascending order of amount).

Condition	Bridged Defect	NBV or NBV/TV	Tb.pf	Cortical Bone	Cancellous Bone (Bone Marrow Cavity)	Fibrotic Tissue
ACS +75 ​μg ​mL^−1^ rhBMP-2 (Positive Control)	+	+++	++	++(+)	+++	+
ColG-FNIII12-14 ​+ ​75 ​μg ​mL^−1^ rhBMP-2	+	+++	+++	+++	+++	–
ColG-FNIII12-14 ​+ ​5 ​μg ​mL^−1^ rhBMP-2	–	+	++	++	++	+
ColG-FNIII12-14 ​+ ​2.5 ​μg ​mL^−1^ rhBMP-2	–	+	+	++	++	+(+)
Plain ACS (Negative Control)	–	+	+	+	+	+++

Histological analysis (the results are summarized in Table 1), which was performed to assess cell infiltration and cell morphology in the area of the defect within the implant, supported the findings obtained from the μCT images. Longitudinal sections of mouse forearms embedded in paraffin were stained with hematoxylin (to stain the nuclei in purplish blue) and eosin (to stain the cytoplasm and extracellular matrix in pink) (Fig. 5 D). The middle section of the implant was chosen from the samples which best represented the average new bone volume in each experimental condition.

In the plain ACS (negative control), cortical bone with a formed bone marrow cavity (maroon and white arrows, Fig. 5 E) could be seen infiltrating the implant space in both the proximal and distal ends, which confirms the results observed in the μCT scans. However, lighter-colored tissue with a more elongated fibroblast-like cell morphology appears in a large area in the remaining gap within the bone defect, suggesting fibrotic tissue formation (black arrows Fig. 5 E).

In the ColG-FNIII12-14 ​+ ​5 and 2.5 ​μg ​mL^−1^ (96.25 and 192.5 ​nM) rhBMP-2 conditions, cortical bone with a fully formed bone marrow cavity could be seen infiltrating deep into the implant area (maroon and white arrows Fig. 5 E) confirming the observations of the μCT scans. In contrast to the plain ACS (negative control), darker in color, bone-like tissue was seen more widely spread throughout the implant. In the ColG-FNIII12-14 ​+ ​5 ​μg ​mL^−1^ (192.5 ​nM) rhBMP-2 condition, a thin sheet of dark pink tissue (cortical bone – maroon arrows, Fig. 5 E) could be seen almost along the full length of the implant. The cell morphology of the tissue in the central area of the implant indicates the formation of a bone marrow cavity. The 2.5 ​μg ​mL^−1^ (96.25 ​nM) rhBMP-2 ​+ ​protein fragment condition also had darker areas (osseous or immature osseous tissue – maroon and white arrows, Fig. 5 E) in the middle of the implant with just a small area of fibrous-looking tissue appearing.

Lastly, in the ACS +75 ​μg ​mL^−1^ rhBMP-2 (positive control) and 75 ​μg ​mL^−1^ (2.88 ​μM) rhBMP-2 ​+ ​protein fragment condition (particularly in the latter) cortical bone (maroon arrows) could be seen throughout the whole implant confirming that the defect was bridged. It appears that the condition with the ColG-FNIII12-14 protein fragment does demonstrate higher amounts of cortical bone compared to the ACS +75 ​μg ​mL^−1^ rhBMP-2 (positive control) condition. Structures that resemble the bone marrow can also be observed in both conditions (white arrows). However, the ACS +75 ​μg ​mL^−1^ rhBMP-2 (positive control) condition exhibited the formation of more fibrotic tissue compared to the protein fragment treated ACS with 75 ​μg ​mL^−1^ rhBMP-2. These findings demonstrate that the ColG-FNIII12-14 and rhBMP-2 treated ACSs have osteogenic potential *in vivo.*

## 3 Discussion

The InFUSE bone regeneration system is composed of an ACS loaded with rhBMP-2 at a concentration of 1.5 ​mg ​mL^−1^ that received FDA approval for clinical application and has been extensively used for multiple applications [2]. However, reducing the dose of rhBMP-2 used while still achieving a therapeutic effect is of primary importance to tackle the safety concerns of the system such as ectopic bone formation, tumorigenesis and nerve damage [[3], [4], [5]].

Taking inspiration from other ECM proteins which bind GFs, as well as collagen binding proteins such as collagenases, we developed a dual affinity protein fragment which can bind both collagen and rhBMP-2. A protein fragment (ColG-FNIII12-14) linking the collagen binding domain of collagenase G. The ColG-FNIII12-14 protein fragment was found to bind strongly to the ACS substrate and can control the release of rhBMP-2 from the ACS substrate.

We also demonstrated that protein fragment treated ACSs show excellent cell attachment, viability and morphology properties which are important to make continue exploring the material *in vitro* worthwhile*.* ColG-FNIII12-14 treated ACSs loaded with rhBMP-2 showed great osteogenesis potential (*in vitro*) when looking at early (Runx2) as well as late osteogenic markers (OPN, mineralization).

ACSs treated with 250 ​μg ​mL^−1^ (4 μΜ) of ColG-FNIII12-14 were also loaded with 75, 5, and 2.5 ​μg ​mL^−1^ (2.88 ​μM, 192.5 ​nM and 96.25 ​nM) of rhBMP-2 and implanted into a non-healing radial defect murine model. A non-ColG-FNIII12-14 treated ACS loaded with 75 ​μg/mL (2.88 ​μM) was used as a positive control while a plain ACS as negative control. The rhBMP-2 concentrations of 75 and 5 ​μg ​mL^−1^ (2.88 ​μM and 192.5 ​nM) were used as the high and low rhBMP-2 concentration limits in our lab in the past [11] while 2.5 ​μg ​mL^−1^ (96.25 ​nM) is the lowest concentration of rhBMP-2 that has been successfully used in a mouse model to date (albeit a different defect model) [6]. To further contextualize the amount of rhBMP-2 used here, other studies using the same radial defect model used 20, 40 and 200 ​μg ​mL^−1^ (770 ​nM, 1.5 ​μM and 7.7 ​μM) of rhBMP-2 as the low, medium and high condition respectively in conjunction with GFOGER-PEG gels [34]. Zwingenberger et al., loaded 167 ​μg ​mL^−1^ (6.4 ​μM) (low dose) and 667 ​μg ​mL^−1^ (25.7 ​μM) (high dose) of rhBMP-2 onto a heparinized collagen substrate to showcase bone regeneration [35]. PEG-fibrinogen substrates have also been loaded with rhBMP-2 (8 ​μg ​mL^−1^ – 308 ​nM) to assess bone regeneration with encouraging results [36]. Similarly, PLGA microspheres loaded with rhBMP-2 showed promising *in vivo* osteogenesis when loaded into brushite, PLGA or more complex mineral, polymer multi-compartment carriers in both rabbit and rat models. The total rhBMP-2 used was (3.5 or 17.5 ​μg) and (1.6 or 6.5 ​μg) respectively [[37], [38], [39]].

In this study, all conditions showed some new bone formation with the ACSs loaded with 75 ​μg ​mL^−1^ (2.88 ​μM), with and without the ColG-FNIII12-14 protein fragment, bridging the gap fully in some samples. The amount of NBV was found to be between 0.5 and 0.6 ​mm^3^ which is comparable to the results shown previously in our lab when using fibronectin-PEG gels, where fibronectin can bind the rhBMP-2 with its heparin binding domain [11]. The amount of NBV decreased when lower amounts of rhBMP-2 were used. ColG-FNIII12-14 treated sponges loaded with 5 and 2.5 ​μg ​mL^−1^ (96.25 and 192.5 ​nM) showed a NBV of 0.22 ​mm^3^ and 0.19 ​mm^3^ respectively which was similar to the NBV measured by Trujillo et al. [11]for similar rhBMP-2 concentrations using the fibronectin-PEG gels. The NBV seen is higher compared to the one showcased by Shekaran et al. where collagen scaffolds loaded with 20 ​μg ​mL^−1^ (770 ​nM) rhBMP-2 showed less than 0.1 ​mm^3^ NBV in the same defect model. The NBV found was also higher compared to the plain ACS (negative control) (0.16 ​mm^3^) albeit the difference was not statistically significant.

Histology studies showed the formation of a bone marrow cavity as well as cortical bone growth in all conditions confirming the results seen in the μCT scans. However, the plain ACS (negative control) showed the formation of fibrotic tissue which was not present in the other conditions to the same extent. Thus, the osteogenic potential of the ColG-FNIII12-14 protein fragment functionalized ACSs loaded with rhBMP-2 concentrations as low as 2.5 ​μg ​mL^−1^ is higher compared to plain ACSs (negative control). Further, the formation of cortical bone and a bone marrow cavity is substantially more evident compared to the histology images by Shekaran et al. who used 20 ​μg ​mL^−1^ (770 ​nM) of rhBMP-2 in the same radial defect model suggesting that functionalizing ACSs with ColG-FNIII12-14 protein fragments before loading them with rhBMP-2 achieves effective and efficient bone regeneration. Nevertheless, rhBMP-2 concentrations between 75 and 5 ​μg ​mL^−1^ (2.88 ​μM and 192.5 ​nM) should be explored to optimize the initial amount loaded, which still achieves defect bridging.

## 4 Conclusion

Taking inspiration from ECM proteins such as fibronectin, we developed dual affinity protein fragments which bind both rhBMP-2 and collagen. These protein fragments were used to treat ACSs similar to the ones used in clinical settings in order to limit the amount of rhBMP-2 necessary for bone regeneration. The ColG-FNIII12-14 protein fragment showed the highest binding potential to the ACS and allowed for increased rhBMP-2 binding and retention compared to plain ACSs. ColG-FNIII12-14 treated ACSs also showed increased osteogenesis *in vitro* after the addition of rhBMP-2. Non-ColG-FNIII12-14 treated ACSs did not showcase the same osteogenic potential despite the addition of rhBMP-2. Lastly, protein fragment treated ACSs with varying amounts of rhBMP-2 were implanted in a critical size defect mouse model, bridging the gap successfully for rhBMP-2 concentrations as low as 2.88 ​μM.

## 5 Experimental section

### 5.1 ACS production

ACSs were produced similarly to the process described by O'Brien et al. [19]. A collagen slurry was prepared by mixing 1% w/w collagen type I bovine tendon powder (Collagen Solutions, UK) with 0.05 ​M acetic acid (Fisher Scientific, USA) solution (pH – 3.2) and briefly (1 ​min) homogenizing it with an T25 Ultra-Turrax from IKA (IKA GmbH, Germany). The collagen mixture was then left to hydrate overnight at 4 ​°C and was homogenized into a slurry a further 5 ​min on ice. Consequently, the slurry was degassed and 150 ​μL was transferred to a mold. The collagen slurry filled molds were then transferred to a freeze-dryer and frozen to −25 ​°C at a rate of −1 ​°C min^−1^. Subsequently the frozen samples were lyophilized at 0 ​°C for 16 ​h and a further 8 ​h at 20 ​°C. The pressure was kept below 50 ​μbar and temperature changes were conducted at 1 ​°C min^−1^. After freeze-drying the samples were crosslinked using EDAC-NHS (Sigma-Aldrich, USA) at a molar ratio of 4:1.6:0.8 (EDAC:NHS:COOH (available free carboxyl groups in collagen)), washed and freeze-dried a second time.

### 5.2 Scanning electron microscopy (SEM)

Material parameters such as mineral and pore size was analyzed through images taken using an FEI Inspect F-50 FE-SEM operated at 10 ​kV (FEI, USA). Sample conductivity was improved through sputter-coating a 4 ​nm layer of platinum using a Leica Microsystems EM ACE600 sputter coater (Leica Microsystems, Germany). The EDX feature of the SEM was also used to create maps of the elements composing the materials produced. The average crystal size and pore size was determined using a MATLAB (The MathWorks Inc., USA) script.

### 5.3 Swelling of ACSs

ACSs were weighed and immersed in milliQ water for 24 ​h. The weight of the hydrated sponge and the weight after water was squeezed out of the pores was also measured. Swelling was assessed using different equations:

Equation (1) $$Swelling\;1\;\left(\%\right)\;\left(total\;swelling\right)=\frac{\left(Weight\;of\;wet\;sponge-Weight\;of\;dry\;sponge\right)\;x\;100}{Weight\;of\;dry\;sponge}$$

Equation (2) $$Swelling\;2\;\left(\%\right)\;\left(fibre\;swelling\right)=\frac{\left(Weight\;of\;squeezed\;sponge-Weight\;of\;dry\;sponge\right)\;x\;100}{Weight\;of\;dry\;sponge}$$

Equation (3) $$Water\;in\;Pores\;\left(\%\right)=\left|\frac{\left(Weight\;of\;squeezed\;sponge-Weight\;of\;dry\;sponge\right)\;x\;100}{Weight\;of\;wet\;sponge}\right|$$

### 5.4 Protein fragment expression and purification in bacteria

The DNA sequence encoding the protein or protein fragment of interest was obtained from the NCBI database. The recombinant proteins were expressed using the pET100/D-TOPO plasmid (ThermoFisher, USA), which features a 6× histidine tag in the N-terminal end of the CDS fused in frame to our sequence, to allow immobilized metal affinity column purification.

DNA plasmids were maintained and purified from NEB 5-alpha strain (New England Biolabs, USA), grown and purified using the Qiagen Miniprep spin kit (Qiagen, Germany). The purified plasmids were transformed in chemically competent BL21 star (DE3) (New England Biolabs, USA) bacteria. Transformed bacteria were cultured in 500 ​mL of previously autoclaved autoinduction media (35.86 ​g ​L^−1^ in ultrapure water) (Formedium Ltd, UK) containing 100 ​μg/mL of ampicillin (Sigma-Adrich, USA) at 37 ^ο^C, 200 ​rpm for 16h. At the end of the culture, the bacterial pellets were spun down at 7000 ​g for 5 ​min in a tabletop centrifuge. The supernatant was discarded, and the pellets frozen at −25 ​°C. The frozen pellets were resuspended in 2 ​mL lysis buffer (0.3 ​mL 1 ​M KH_2_PO_4_, 4.7 ​mL 1 ​M K_2_HPO_4_, 2.3g NaCl, 0.75g KCl, 10 ​mL glycerol, 0.5 ​mL Triton ×100, 68 ​mg Imidazole, ultrapure water and HCl to adjust pH to 7.8100 ​mL (Sigma-Aldrich, USA)) supplemented with complete protease inhibitor cocktail according to manufacturer's instruction (Roche, Switzerland). The resuspended bacteria were sonicated, using a 5 ​s on, 5 ​s off duty cycles for a total of 10 ​min in ice. The lysate was centrifuged at 20,000 ​rpm for 40 ​min and filtered to remove the debris. The Akta Start (Cytiva, USA) system containing a HisTrap nickel column was used for purification. The purified fractions containing the recombinant protein were dialyzed against PBS (pH 7.4), sterile filtered and stored at −80 ​°C. Protein size and purity was verified using SDS-PAGE with a total protein staining using Coomassie brilliant blue (Bio-rad, USA).

### 5.5 Protein fragment absorption-release

To assess the absorption and release of the ColG-FNIII(12–14), the ColH-FNIII(12–14) and PLGF-FNIII(12–14) from collagen, collagen scaffolds were treated with 75 ​μg of each protein fragmenting PBS for 1h at room temperature. An aliquot of the protein fragment solution pre- and post-incubation with collagen was taken and frozen at −20 ​°C. To assess the release of protein fragments from collagen substrates they submerged in 150 ​μL of PBS for up to 5 days at 37 ​°C. The supernatant was removed at 36, 60, 120 ​h and frozen at −20 ​°C and fresh PBS was added. To measure the protein fragment concentration of the supernatant during release as well as pre- and post-absorption, a micro bicinchoninic acid protein assay kit (Micro BCA® (ThermoFisher, USA) was used. Briefly, 150 ​μL of standards and samples were loaded into a 96 well plate and mixed with the same volume of working solution (25:24:1 (V:V:V) of Reagents A:B:C). The well plate was sealed and incubated for 2h at 37 ​°C protected from light. After incubation, the absorbance at 562 ​nm was measured using a plate reader. The absorption and cumulative release of each protein fragment was calculated as a percentage to the initial amount of protein fragment used to treat the collagen substrates.

The same procedure was carried out to assess the impact of varying the amount (75, 37.5 and 15 ​μg) of ColG-FNIII12-14 used to treat collagen scaffolds.

### 5.6 Protein fragment immunofluorescence

ACSs were incubated with 37.5 ​μg of ColG-FNIII12-14 in PBS for 1 ​h at room temperature. Samples were blocked with a blocking buffer (1% BSA in PBS) for 30 ​min at room temperature. An anti his-tag monoclonal mouse antibody (Invitrogen, USA) in blocking buffer was added at a concentration of 1:1000 and incubated for 1 ​h at room temperature. Samples were washed 4 times for 5 ​min with wash buffer (0.5% Tween buffer) before the addition of goat-anti-mouse Cy3 secondary antibodies (Jackson Immunoresearch, USA) were added at 1:200 and incubated for 1 ​h at room temperature in the dark. The samples were washed 4 more times for 5 ​min with wash buffer before images were taken using a Zeiss AxioObserver Z1 (Zeiss, Germany) at varying magnifications (10×, 20× and 40×).

### 5.7 hMSC culture

hMSCs (PromoCell, Germany) were seeded on cell culture flasks at a cell density of 5000 ​cells/cm^2^ in 20% fast growth and 80% regular media (DMEM supplemented with 1% antibiotic mix (fungizone, penicillin/streptomycin), 1% sodium pyruvate, 1% NEAA and 10% FBS) (Gibco, USA). The cell medium was changed on the third day to 100% regular media and the cells grown until 80% confluency before further use at 37 ​°C and 5% CO_2_. Regular cell media was refreshed every two-three days.

### 5.8 Cell viability assays (LIVE/DEAD)

Cells (hMSCs) were seeded at a density of 3.5 ​× ​10^5^ ​cells/mL for up to 6 days on protein fragment treated collagen scaffolds. On day 2 and day 6 scaffolds were stained after incubating them for 30 ​min at 37 ​°C in PBS with calcein (1:200) and ethidium homodimer-1 (1:1000) (ThermoFisher, USA). The substrates were washed 3× for 5 ​min with PBS and imaged using a Zeiss fluorescence microscope (Zeiss AxioObserver Z1 (Zeiss, Germany)). Live cells appeared on the green channel (excitation wavelength 494 ​nm, emission wavelength 517 ​nm) and dead cells on the red channel (excitation wavelength 517 ​nm, emission wavelength 617 ​nm). Viability was calculated as a percentage of live to dead cells.

### 5.9 Cell immunostaining protocol

Cells were fixed using 4% paraformaldehyde for 15 ​min at RT, permeabilized for 5 ​min using 0.1% TritonX100 (Sigma-Aldrich, USA) and blocked with 1% BSA (Sigma-Aldrich, USA). Cells were incubated in 1% BSA with a primary for 1 ​h at RT and washed 4 times for 5 ​min with wash buffer (0.5% Tween20 (Sigma-Aldrich, USA)). The secondary antibody and phalloidin was added for 1h at RT (protected from light) before the sample was washed a further 4 times for 5 ​min. NucBlue (ThermoFisher, USA) at 1 drop mL^−1^ was added and the samples were incubated for 20 ​min at RT. Samples were briefly washed again (2 ​× ​5 ​min) and imaged using a Zeiss AxioObserver Z1 (Zeiss, Germany). Image analysis was carried out in ImageJ.

### 5.10 Cell adhesion and morphology scaffolds

Cells (hMSCs) were seeded at a density of 3.5 ​× ​10^4^ ​cells mL^−1^ for 4 ​h on protein fragment treated collagen scaffolds at 37 ​°C and 5% CO_2_ without FBS. Cells were stained for vinculin (primary anti vinculin mouse monoclonal antibody 1:500 (ThermoFisher, USA) with a secondary rabbit anti-mouse Cy3 (Jackson Immunoresearch, USA) at 1:200), actin (Alexa Fluor 488 phalloidin 1:100) and the nucleus (NucBlue) following the protocol described above.

### 5.11 Cell osteogenic differentiation studies

To assess the ability of collagen substrates with GF binding protein fragments to induce osteogenesis cells (hMSCs) were seeded and kept in culture (37 ​°C and 5% CO_2_) for up to 28 days on different substrates of interest (ACSs and gels) at a density of 1 ​× ​10^4^ ​cell cm^−2^ (2D), 3.5 ​× ​10^5^ ​cells mL^−1^ (2.5D) and 1 ​× ​10^6^ ​cells mL^−1^ (3D). Samples were imaged using a Zeiss AxioObserver Z1 inverted epifluorescence microscope. Regular hMSC culture media was used for all conditions apart from the positive control for which osteogenic media (regular media, 100 ​nM dexamethasone, 50 ​μM L-ascorbic 2 phosphate and 10 ​mM β-glycerophosphate disodium salt hydrate (Sigma-Aldrich, USA)) was used. *Runx2* translocation to the nucleus was assessed after 6 days of culture through immunostaining for Runx2 (primary anti Runx2 1:400 (Santa Cruz, USA), secondary rabbit-anti-mouse Cy3 (Jackson Immunoresearch, USA) 1:200), actin (Alexa Fluor 488 phalloidin 1:100 (ThermoFisher, USA) and the nucleus (NucBlue 1 drop mL^−1^). Nuclear intensity was measured using ImageJ which was. OPN expression was assessed after 21 days of culture through immunostaining for OPN (primary rabbit-anti-OPN 1:400, goat-anti-rabbit Cy3 (Jackson Immunoresearch, USA) 1:200), actin (Alexa Fluor 488 phalloidin 1:100) and the nucleus (NucBlue 1 drop mL^−1^). Matrix mineralization was assessed through a von Kossa after 28 days. Cultures were washed 3× with PBS and fixed with 4% paraformaldehyde. Cultures were washed again briefly and covered in an aqueous solution of 5% w/w silver nitrate (Sigma-Aldrich, USA). The cultures were exposed to 30 ​min of UV light and rinsed using MilliQ water. A solution of 5% sodium thiosulphate (Sigma-Aldrich, USA) was then added for 5–10 ​min and cultures were again washed with MilliQ water and imaged using a stereoscopic microscope (Zeiss, Germany).

### 5.12 rhBMP-2 labelling

rhBMP-2 (carrier free) (R&D systems, USA) was fluorescently labelled with an amino reactive dye (Dylight® NHS Ester 488 (Thermofisher, USA) following the manufacturer's standard protocol. BMP-2 was re-suspended in 0.05 ​M sodium borate buffer (pH-8) and Dylight 488 dye was added to the solution and incubated for 1 ​h at room temperature in the dark. The amount of dye was calculated based on the following equation:

Equation (4) $$\frac{Amount\;of\;BMP2\;in\;\mu g}{MW\;of\;BMP2}x\;10\;x\;MW\;of\;NHS\;ester$$

Consequently, the solution was dialyzed 4× for 1 ​h against PBS and stored in 10 ​μL aliquots at −20 ​°C until further use.

### 5.13 GF absorption release experiments

To study the absorption of rhBMP-2 on collagen substrates with and without protein fragments, rhBMP-2 was added at 0.1, 0.5, 1 and 2 ​μg ​mL^−1^ and incubated for 1 ​h at 37 ​°C protected from light. Aliquots were taken pre- and post-treatment and their fluorescent intensity was measured in a black 96 well plate using a plate reader (excitation wavelength: 492 ​nm emission wavelength 518 ​nm). The amount of BMP-2 absorbed was calculated as a percentage:

Equation (5) $$BMP2\;absorbed\;\left(\%\right)=\frac{BMP2_{preabsorption}-BMP2_{postabsorption}}{BMP2_{preabsorption}}\;x\;100$$

To assess the release of BMP-2, collagen substrates treated with BMP-2 were submerged in PBS at 37 ​°C for up to 5 days protected from light. Samples from the supernatant were taken at 36, 60, 120 ​h and fresh PBS was added at each time point. The supernatant samples for each condition were again read using a fluorescent plate reader. The release values found at each timepoint was added to the result of the previous timepoint and the cumulative release was calculated as follows:

Equation (6) $$Cummulative\;Release\;\left(\%\right)=\sum_{n=36,60,120}^{120}\frac{BMP2_{release}\left(n\right)\;x\;100}{BMP2_{preabsorption}-BMP2_{postabsorption}}$$

A Monolith NT.115 (NanoTemper GmbH, Germany) MST was used to approximate the Kd of the interaction which was achieved using the NanoTemper Software.

### 5.14 Murine non-healing bone defect model

This experiment was conducted under the Animals (Scientific Procedures) ACT 1986 (ASPel project license n° PP5891831). All the research performed, complied with ethical regulations approved by the University of Glasgow's ethical committee.

### 5.15 Implant preparation

Polyimide implant tubes presenting holes were used as sleeves and filled with 3 ​μL collagen slurry (made as described previously) using a positive displacement pipette. The implants were consequently freeze-dried, crosslinked with EDAC-NHS, washed, and freeze-dried again a second time as described previously. The implants were sterilized with ethylene oxide before further use and treated with protein-fragments and/or BMP-2 the day before implantation by submerging the implants in a solution containing the protein fragments and then rhBMP-2 for 1h at RT. The experimental conditions tested are listed below:

1) ACS with 75 ​μg ​mL^−1^ BMP-2 (i)
2) ACS with 250 ​μg ​mL^−1^ protein fragment (CollagenaseG_s3a-s3b_ ​+ ​FNIII_12-14_) and 75 ​μg ​mL^−1^ BMP-2 (ii)
3) ACS with 250 ​μg ​mL^−1^ protein fragment (CollagenaseG_s3a-s3b_ ​+ ​FNIII_12-14_) and 5 ​μg ​mL^−1^ BMP-2 (iii)
4) ACS with 250 ​μg ​mL^−1^ protein fragment (CollagenaseG_s3a-s3b_ ​+ ​FNIII_12-14_) and 2.5 ​μg ​mL^−1^ BMP-2 (iv)
5) ACS (vi)

### 5.16 Bone radial segmental defect surgery

C57BL/6 male and female mice (8 weeks old, Charles River, USA) were anaesthetized using isoflurane gas and the right forelimb was shaved and swabbed with povidone-iodine. Mice received a dose of buprenorphine and carprofen for pain relief. An incision was performed along the right forearm and the soft tissue over the radius was blunt-dissected using a periosteal elevator to expose the bone. A 2.5 ​mm defect was created in the center of the radius using a custom-made parallel double-bladed bone cutter. Care was taken to leave the ulna intact, and the 4 ​mm length implant was introduced into the radial bone defect, abutting its proximal and distal ends. After repositioning the muscle and skin, the incision was closed with a degradable suture and the mice were monitored for signs of distress, movement, and weight loss. 6 mice (3 males and 3 females) were used per experimental condition.

### 5.17 Analysis of bone growth

Eight weeks post-surgery the mice were sacrificed, and the bone samples were explanted, fixed in 4% para-formaldehyde and immersed in 70% ethanol.

#### 5.17.1 Micro CT

The bone samples were analyzed using microcomputer tomography (μCT, Bruker SkyscanMicro X-ray CT) (Bruker, USA). Bone volume was quantified using the CTAn software (Bruker, USA). To ensure that only the volume of new mineralized bone was measured, a 2.0 ​mm length section, in the middle of the 2.5 ​mm defect, was selected as a Volume of interest (VOI) which has Total Volume (TV) of 1.5 ​mm^3^.

#### 5.17.2 Histological analysis

Bone samples were decalcified using Krajian solution (citric acid, formic acid, from Ricca) for 3 ​d, until soft and pliable. Samples were then embedded in paraffin and sectioned. For histological analysis, sections (of 7 ​μm thickness) were stained for hematoxylin and Eosin (H&E), and alcian blue and picrosirius red staining. Briefly, sections were deparaffinized and rehydrated in water Samples were mounted and imaged with an EVOS FL microscope (ThermoFisher, USA) at 10 ​× ​magnification.

### 5.18 Statistical analysis

Statistical significance (P ​< ​0.05) between groups was showing using analysis of variance (ANOVA) and post-hoc paired t-tests between individual condition in GraphPad Prism 9 (GraphPad Software, USA). The Holm-Sidak method was used to correct for multiple comparisons.

## Credit author statement

**Stylianos O. Sarrigiannidis**: Conceptualisation, methodology, investigation, writing – original draft. **Oana Dobre**: Supervision, methodology, writing – review & editing. **Alexandre Rodrigo Navarro**: Supervision, methodology, writing – review & editing. **Matthew J. Dalby**: Conceptualisation, supervision, funding acquisition, writing - review & editing. **Cristina Gonzalez-Garcia**: methodology, investigation, writing – review & editing. **Manuel Salmeron-Sanchez**: conceptualisation, methodology, supervision, writing – original draft, funding acquisition.

## Declaration of competing interest

The authors declare the following financial interests/personal relationships which may be considered as potential competing interests:Manuel Salmeron-Sanchez reports financial support was provided by 10.13039/501100000853University of Glasgow.

## Data Availability

Data will be made available on request.
