# Supplementary material for: Engineered dual affinity protein fragments to bind collagen and capture growth factors
Source: Mater Today Bio. 2023 Apr 22;20:100641. doi: 10.1016/j.mtbio.2023.100641 (PMC10173277; doi:10.1016/j.mtbio.2023.100641)
Supplement: Multimedia component 1 [file mmc1.docx]

**Engineered dual affinity protein fragments to bind collagen and capture growth factors**

*Stylianos O. Sarrigiannidis, Oana Dobre, Alexandre Rodrigo Navarro, Matthew J. Dalby, Cristina Gonzalez-Garcia, Manuel Salmeron-Sanchez**

Stylianos O. Sarrigiannidis, Oana Dobre, Alexandre Rodrigo Navarro, Matthew J. Dalby, Cristina Gonzalez-Garcia, Manuel Salmeron-Sanchez

Centre for the Cellular Microenvironment, University of Glasgow, Advanced Research Centre (ARC), Glasgow G12 8QQ, United Kingdom

E-mail: [Manuel.Salmeron-Sanchez@glasgow.ac.uk](mailto:Manuel.Salmeron-Sanchez@glasgow.ac.uk)

Keywords: collagen, bone regeneration, recombinant protein fragment, fibronectin

**Supplementary Information:**

*
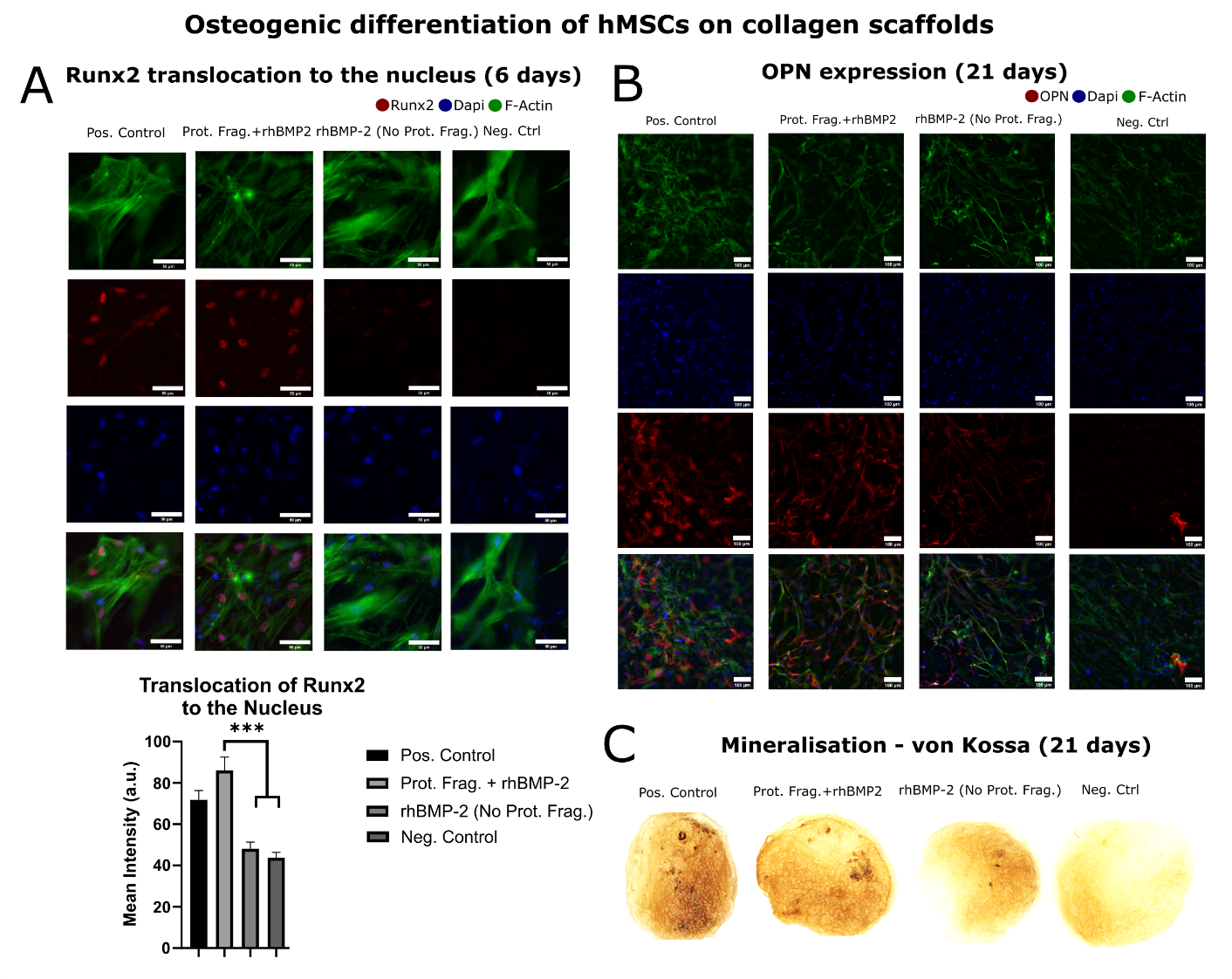

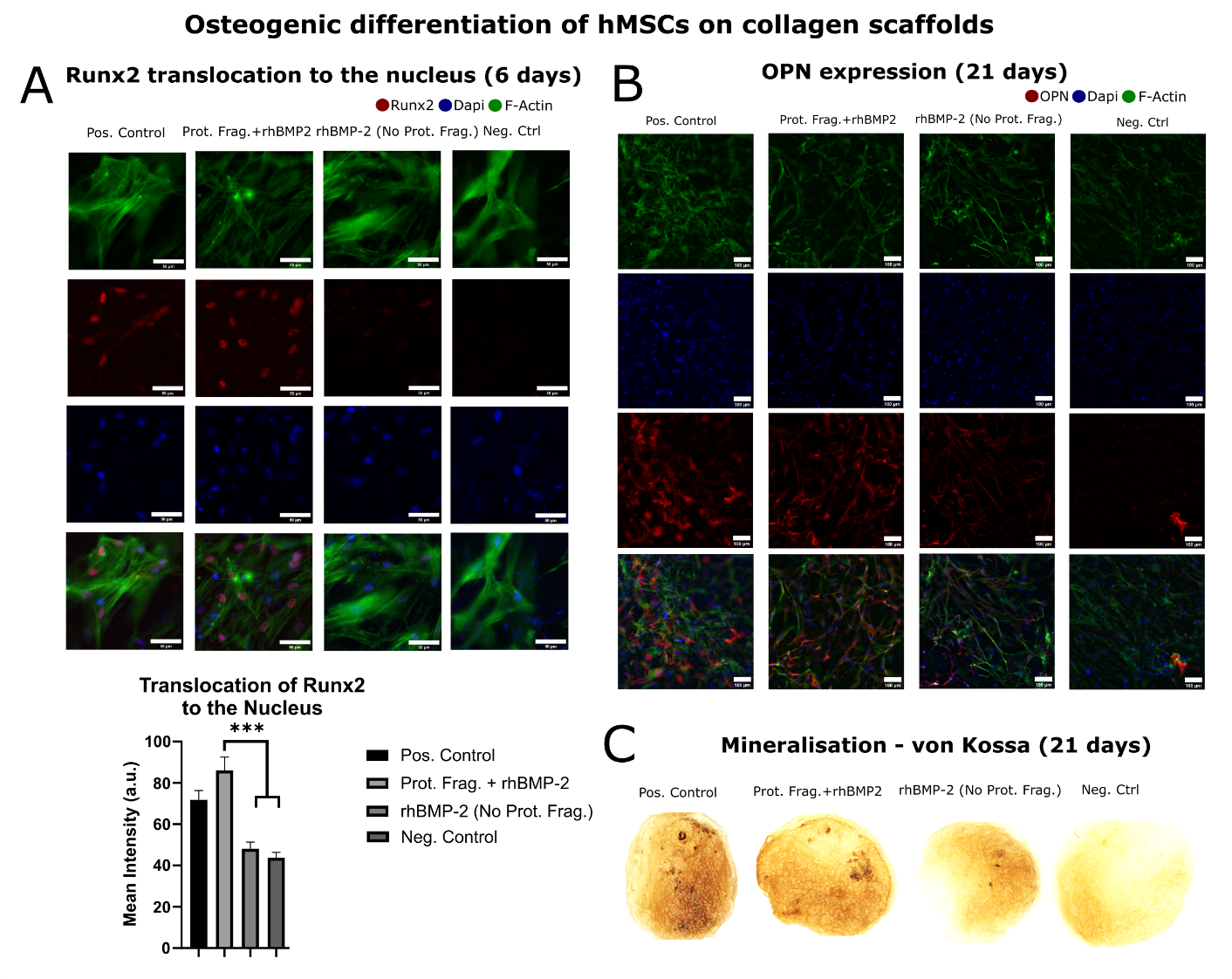
*

Supplementary Figure 0: Larger image of Figure 4A showing fluorescence images of Runx-2, F-actin of hMSCs on collagen sponges after 6 days. Pos. Control (osteogenic media), neg. Control (plain sponge), Scale bar (50μm)

*
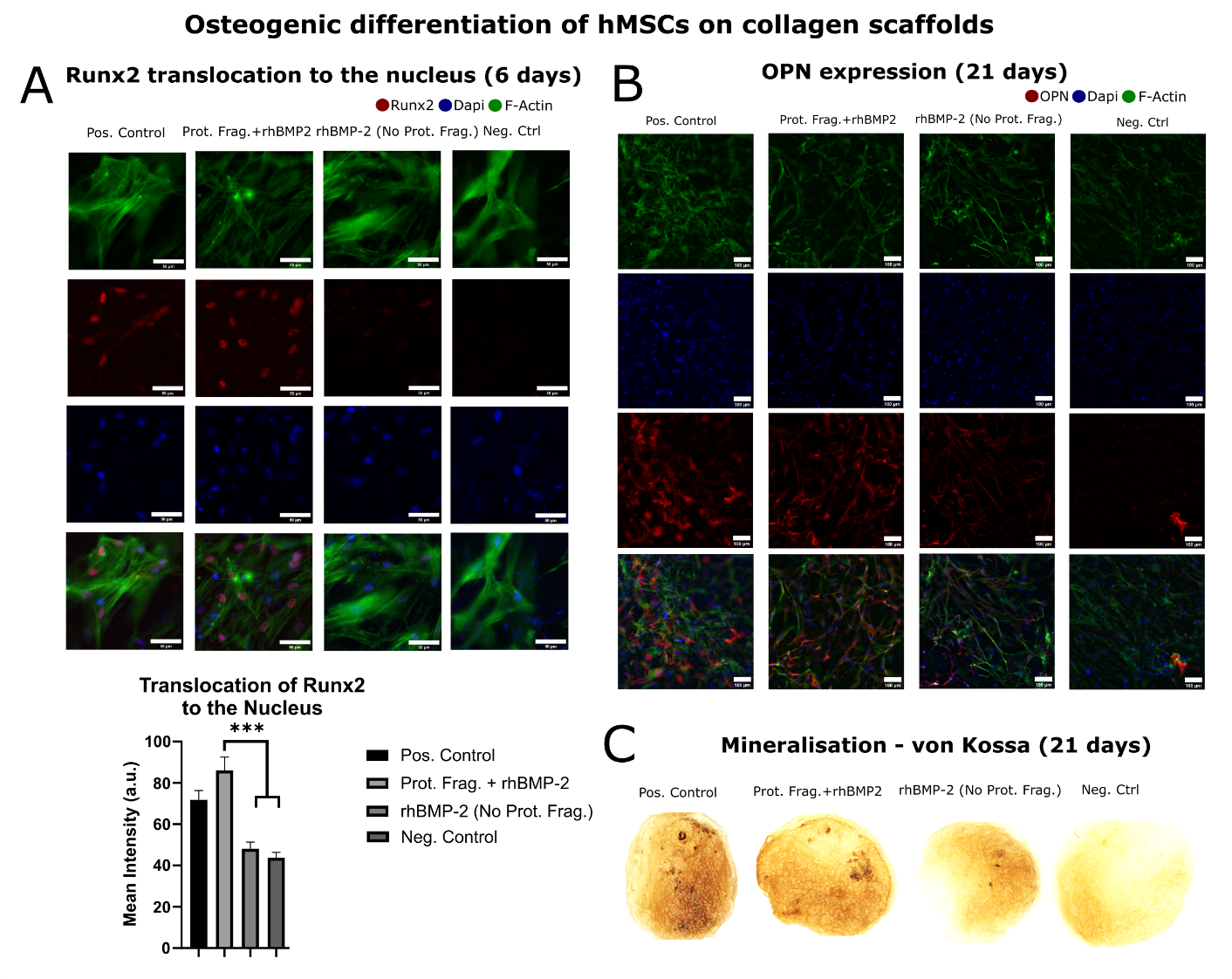
*

*
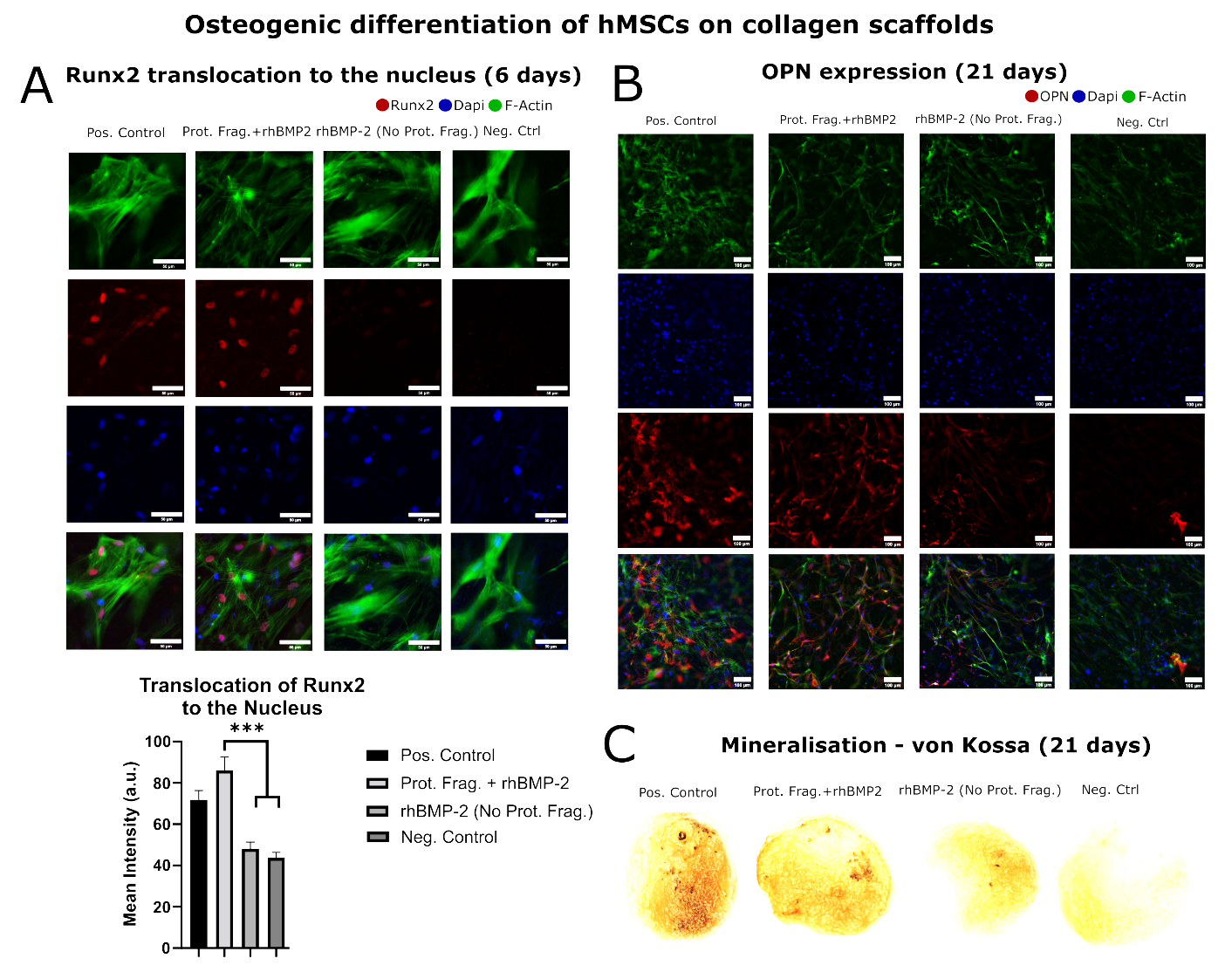
*

*
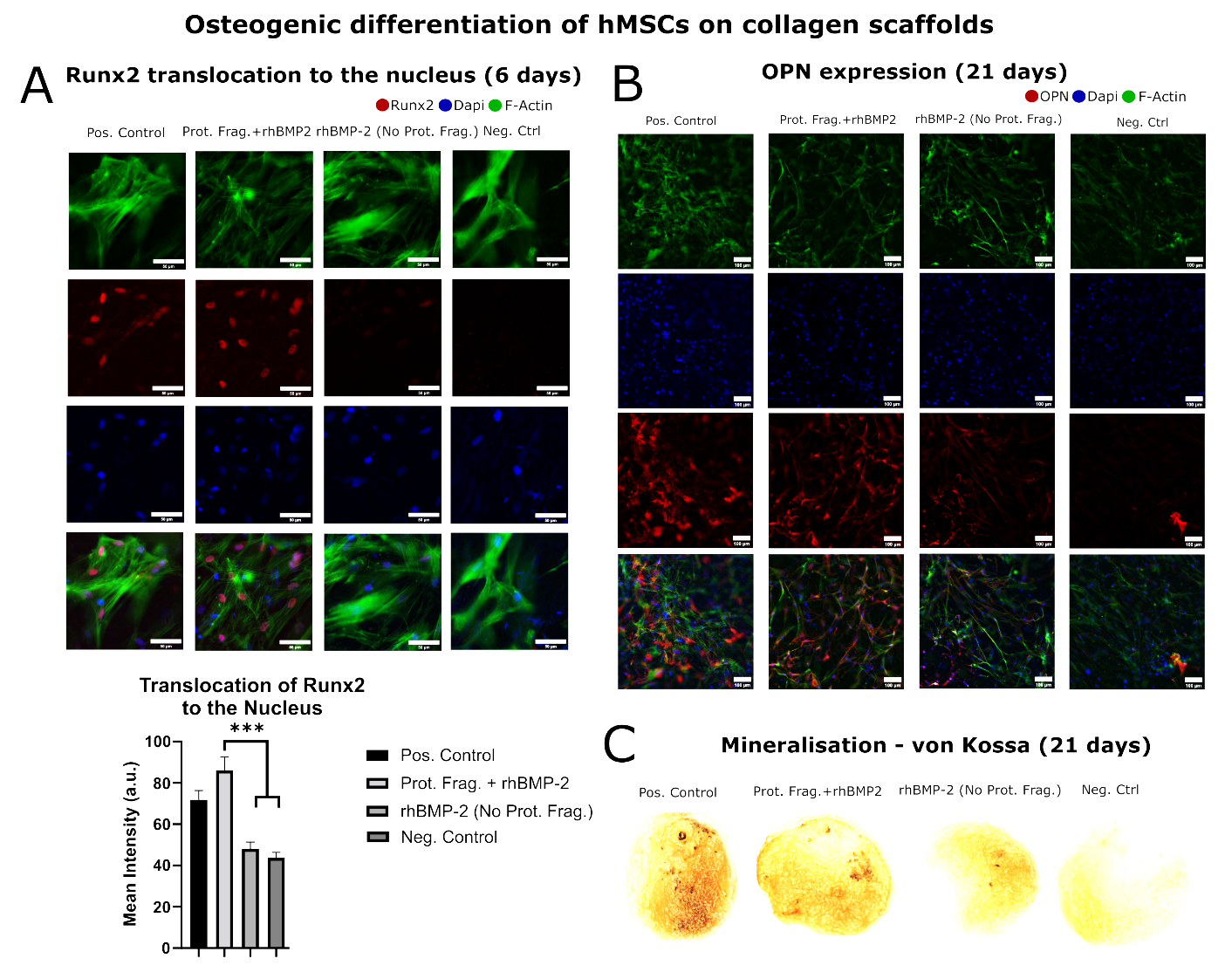
*

Supplementary Figure 1: Larger image of Figure 4B showing fluorescence images of OPN, F-actin and Dapi staining of hMSCs on collagen sponges after 21 days. Pos. Control (osteogenic media), neg. Control (plain sponge), Scale bar (100μm)


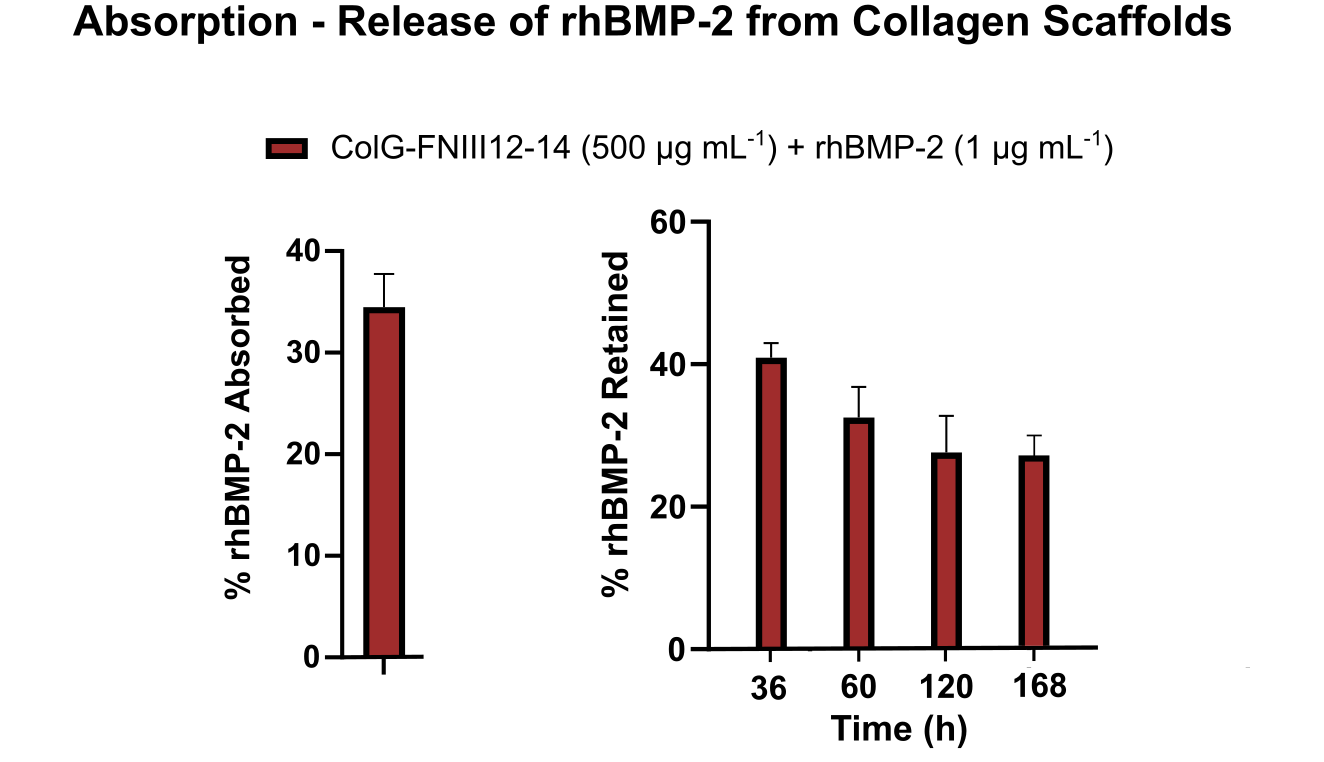


Supplementary Figure 2: Shows the % rhBMP-2 (1 μg mL^-1^) absorbed on collagen scaffolds treated with 500 μg mL^-1^ of ColG-FNIII12-14 protein fragment and compared to rhBMP-2 absorption onto non-protein fragment treated collagen scaffolds. The release of the rhBMP-2 absorbed onto the scaffolds was also monitored for a 168 h period.

**
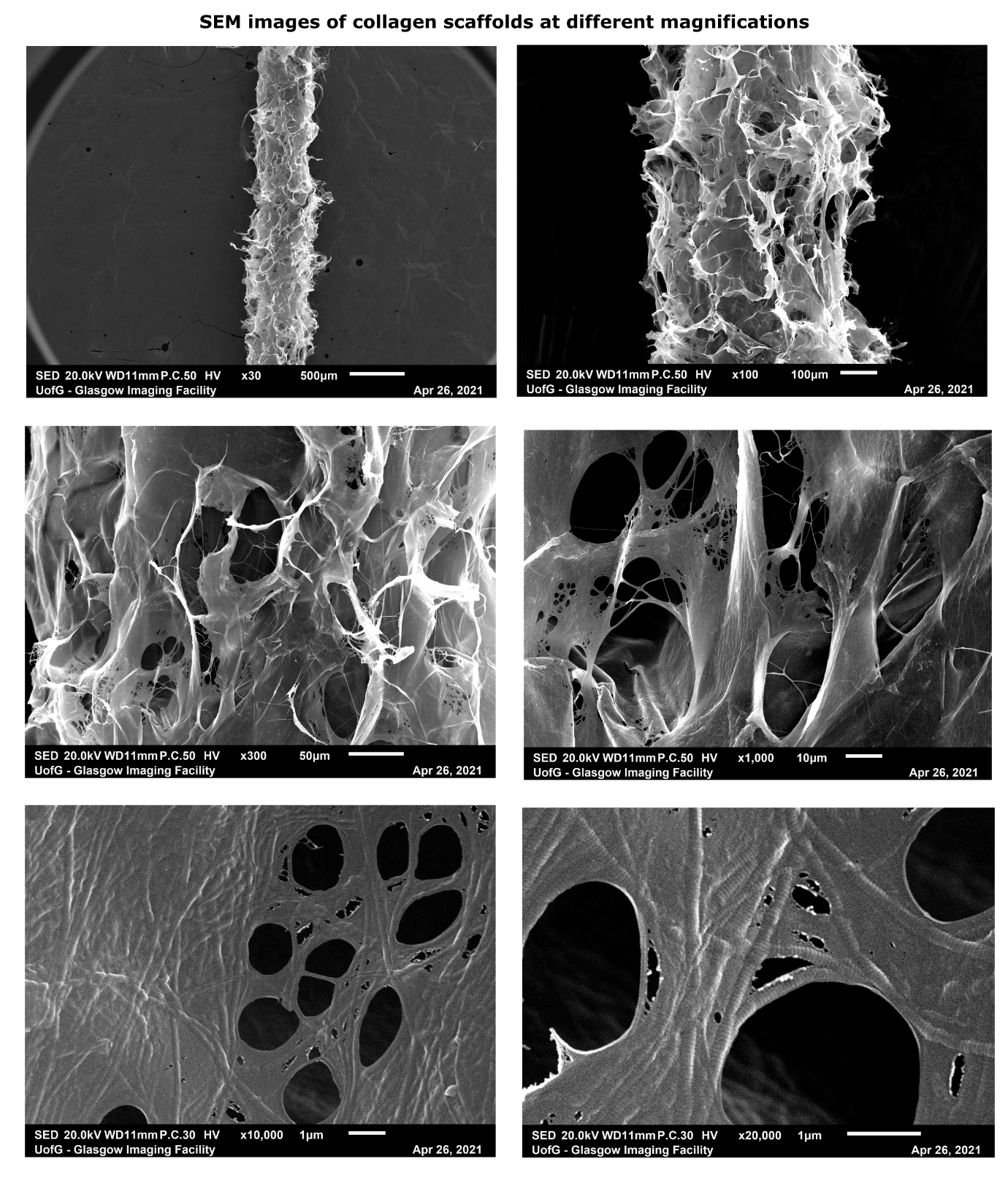
**

1 μm

50 μm

1 μm

10 μm

100 μm

500 μm

Supplementary Figure 3: SEM images of the collagen scaffolds made for in vivo application in mice. The scaffold is depicted at different magnifications. A network of interconnected pores of different sizes can be observed. At very high magnifications (x10.000 and x20.0000) the collagen fibres can be seen with their characteristic striated morphology.


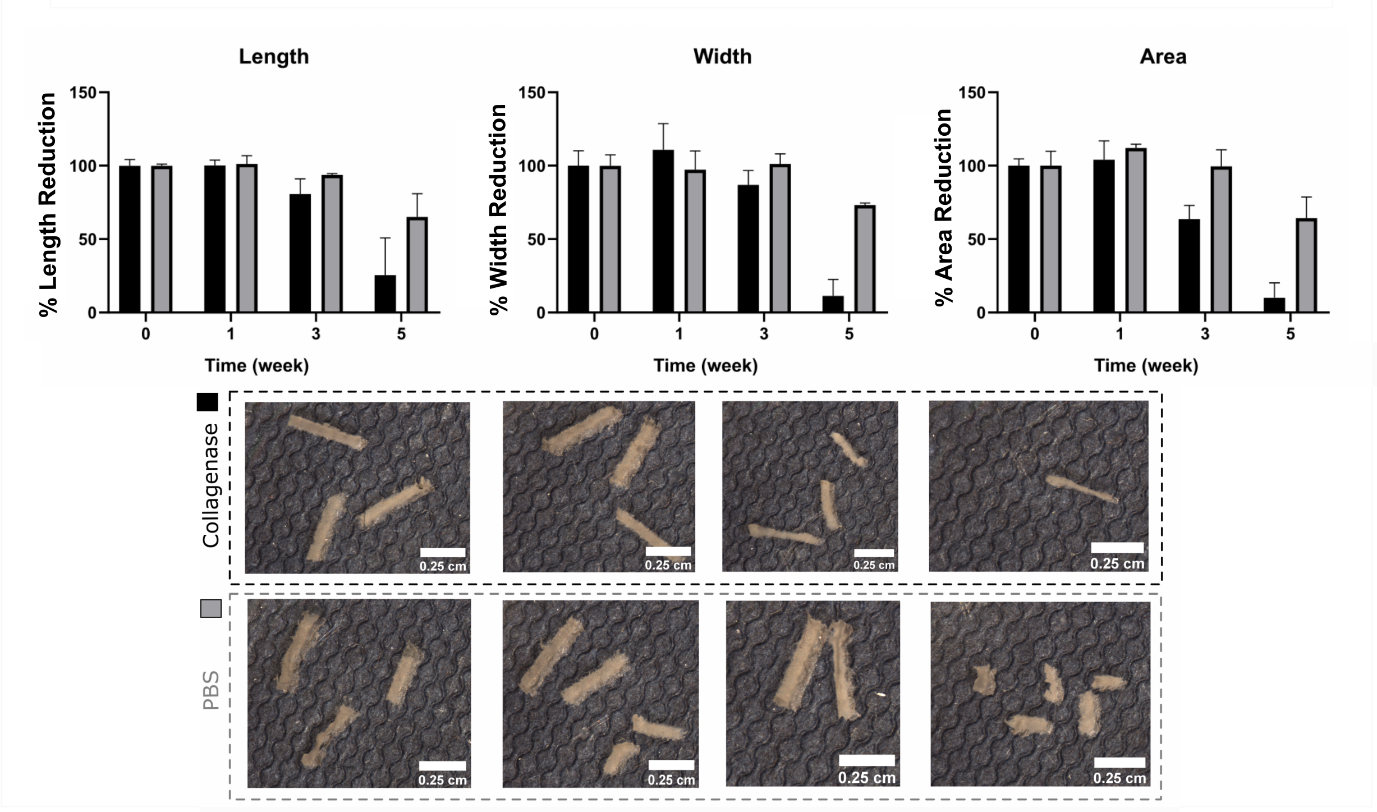


Week 0

Week 1

Week 5

Week 3

Supplementary Figure 4: Degradation studies for collagen scaffolds used for mice in vivo studies carried out in both PBS and 10U/mL collagenase over a period of 5 weeks. It can be clearly seen that the scaffolds degrade both hydrolytically (PBS) and enzymatically (collagenase) over the experimental period. As expected, the degradation in collagenase is quicker than PBS, and the scaffolds are almost completely degraded by week 5. Note that for the last time point two of the collagen sponges in collagenase had fully degraded and the collagen in PBS broke into smaller pieces when removing them from the implant tube to measure. The length, width and area of the pieces removed from the same implant tube was added.


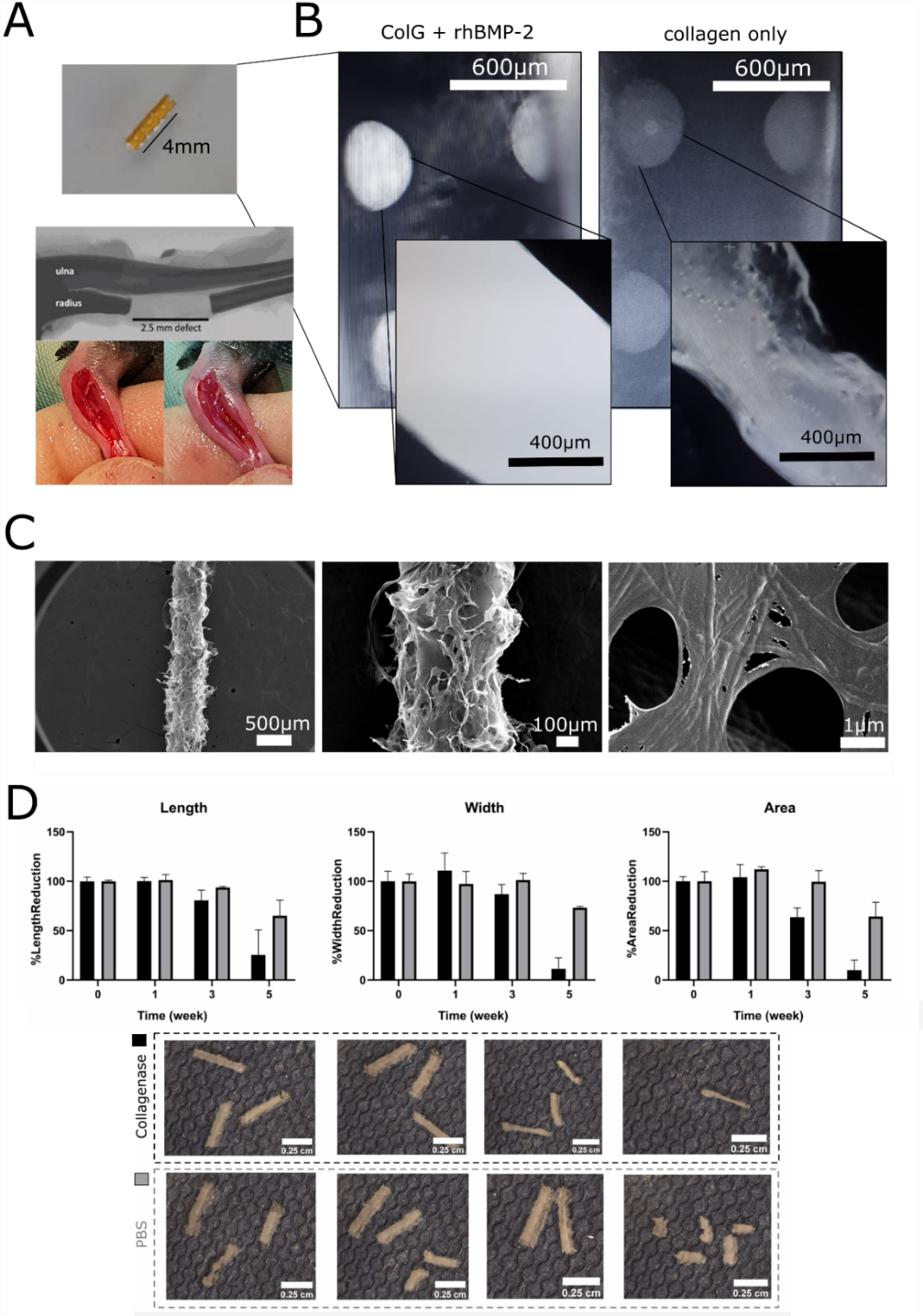


Supplementary Figure 5: A) The collagen sponge within the 4mm implant tube used to implant into a 2.5mm (non-healing) critical size radial mouse bone defect. B) rhBMP-2 solution was absorbed successfully by the ColG treated sponge even when placed within the implant tube as can be seen in the fluorescent microscopy images.


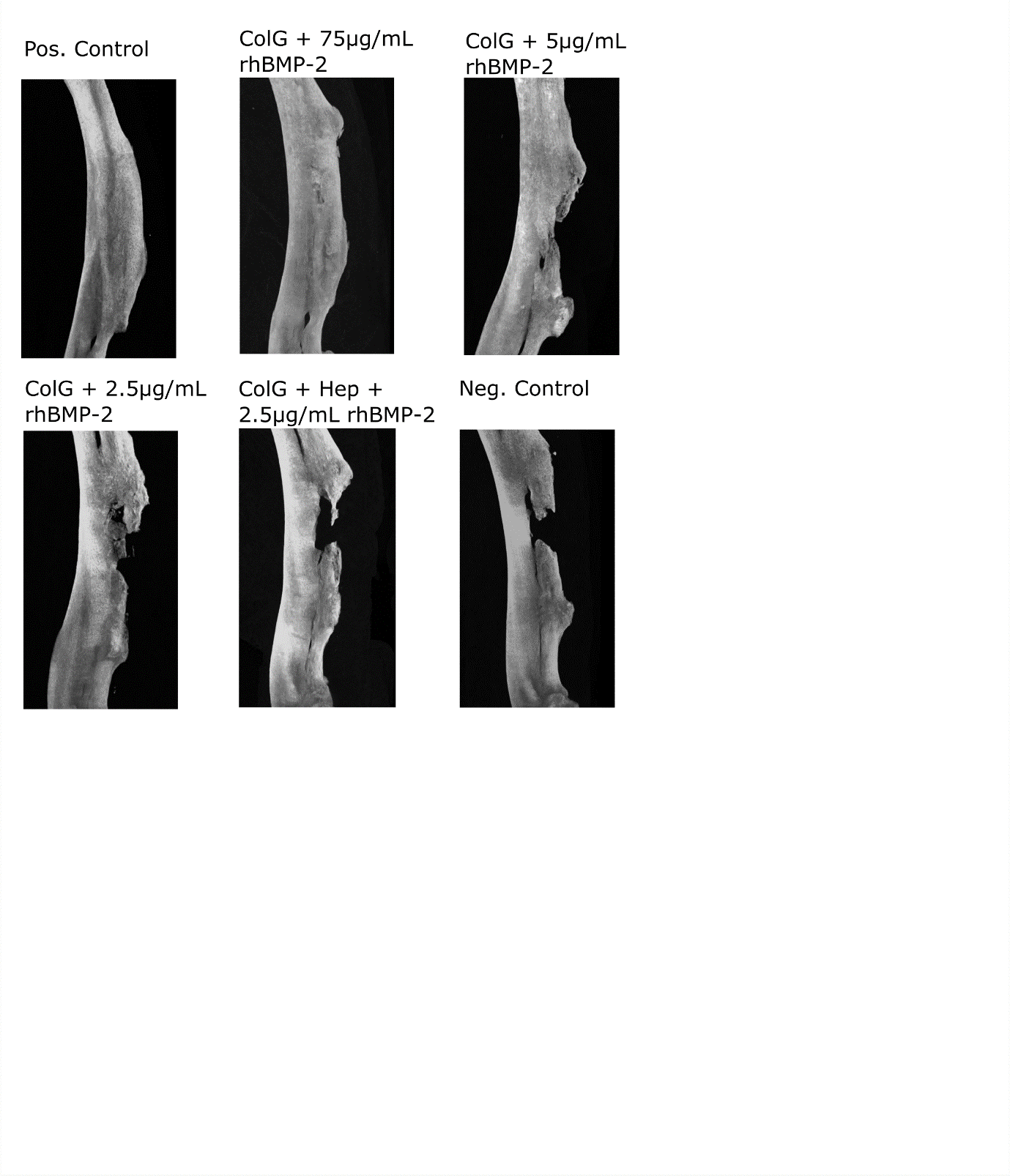


Supplementary Figure 6: 3D reconstructions of μCT images showing the area where the implant was placed in the critical size murine radial defect. The samples depicted showed the highest amount of new bone volume formation in each of the conditions. Pos. Control (75μg mL­_­_^-1^ rhBMP-2), neg. Control (plain sponge)

Supplementary Figure 7: Trabecular bone pattern factor quantified for ACS with 75 μg mL^-1^ rhBMP-2 with no added ColG-FNIII12-14 protein fragment (positive control), 250 μg mL^-1^ ColG-FNIII12-14 + 75 μg mL^-1^ rhBMP-2, 250 μg mL^-1^ ColG-FNIII12-14 + 5 μg mL^-1^ rhBMP-2, 250 μg mL^-1^ ColG-FNIII12-14 + 2.5 μg mL^-1^ rhBMP-2, and plain ACS (negative control) condition (mean +/- SEM, n=6, Statistical Significance: * p <0.05.


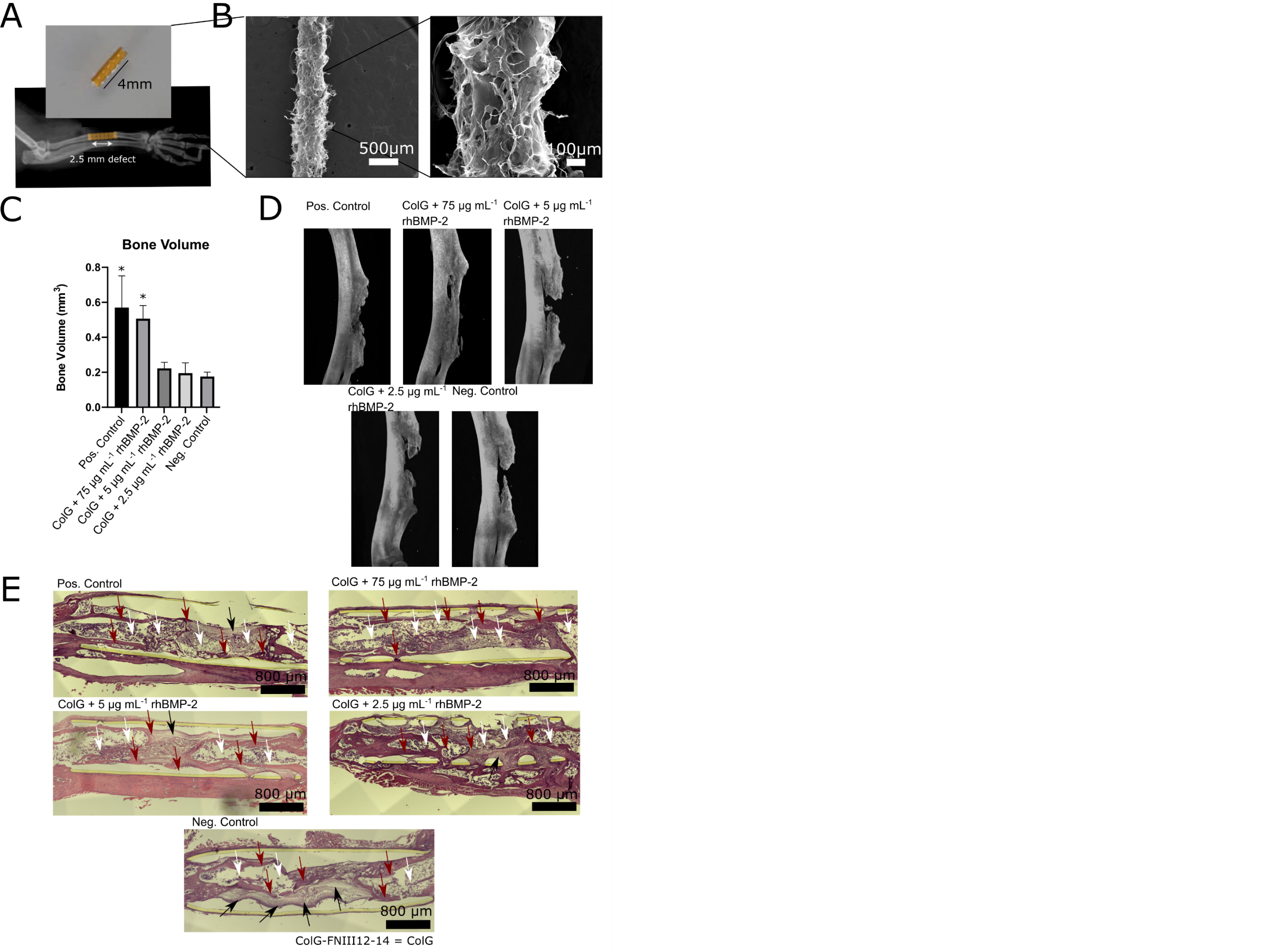

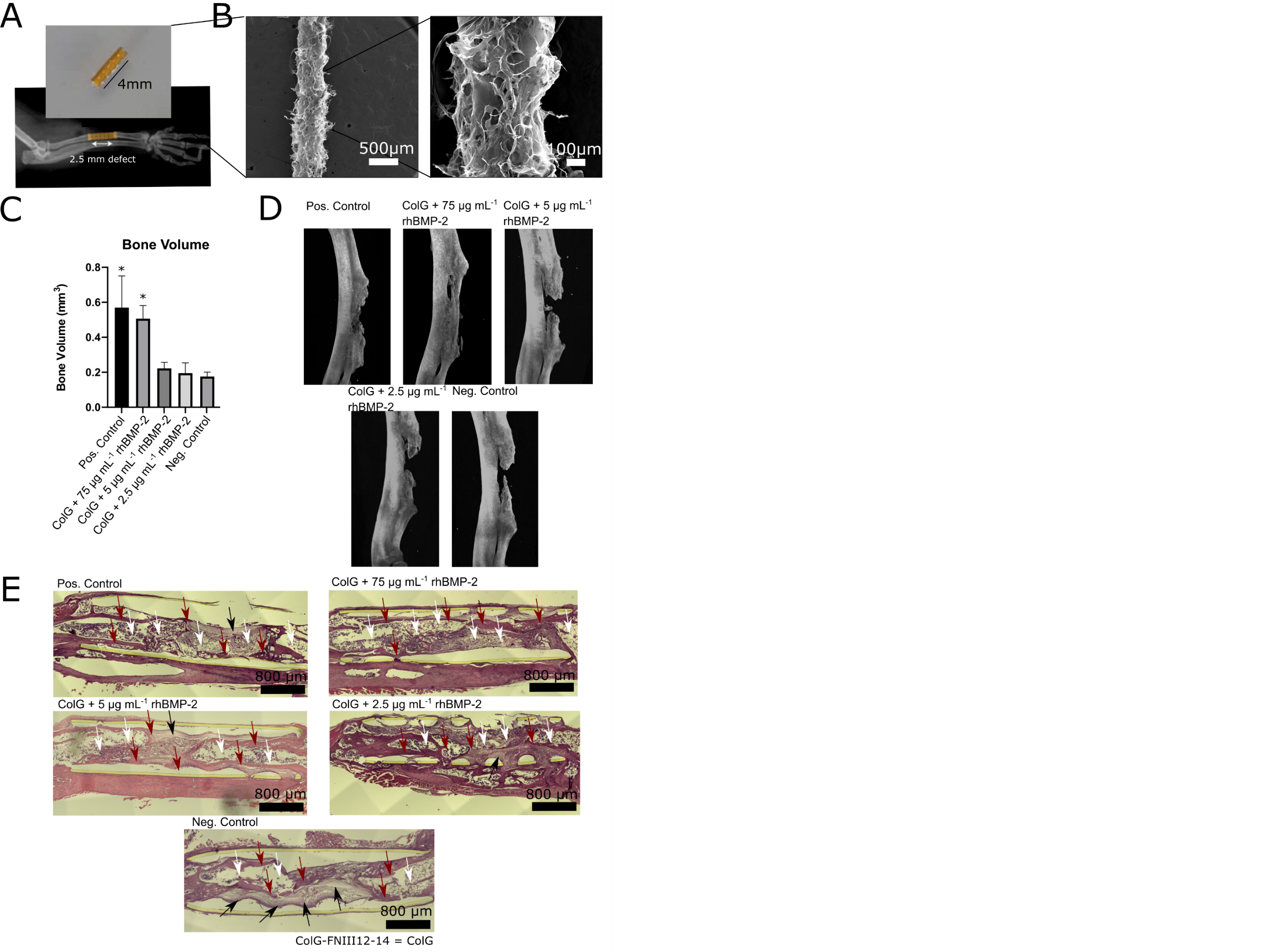


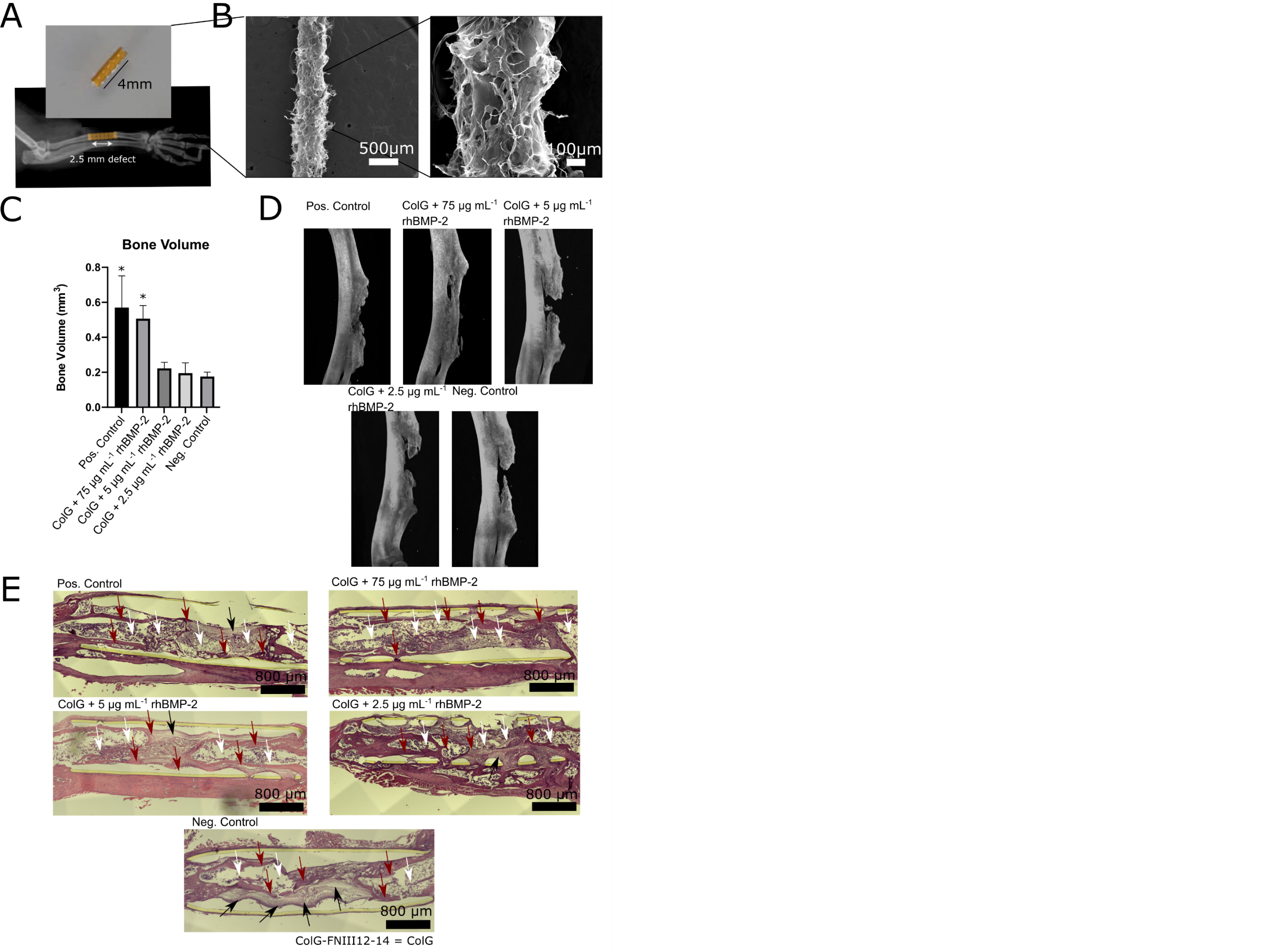


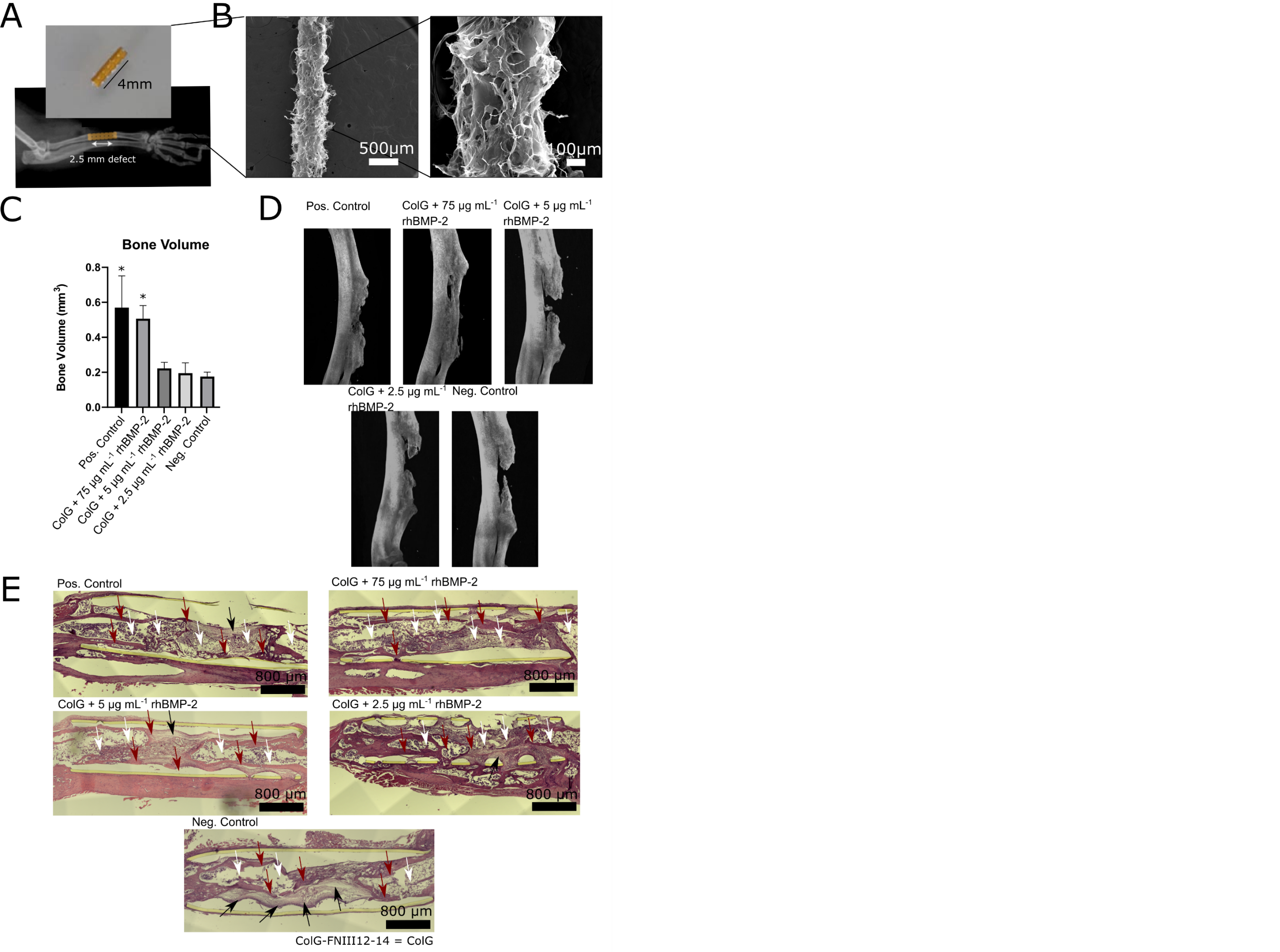


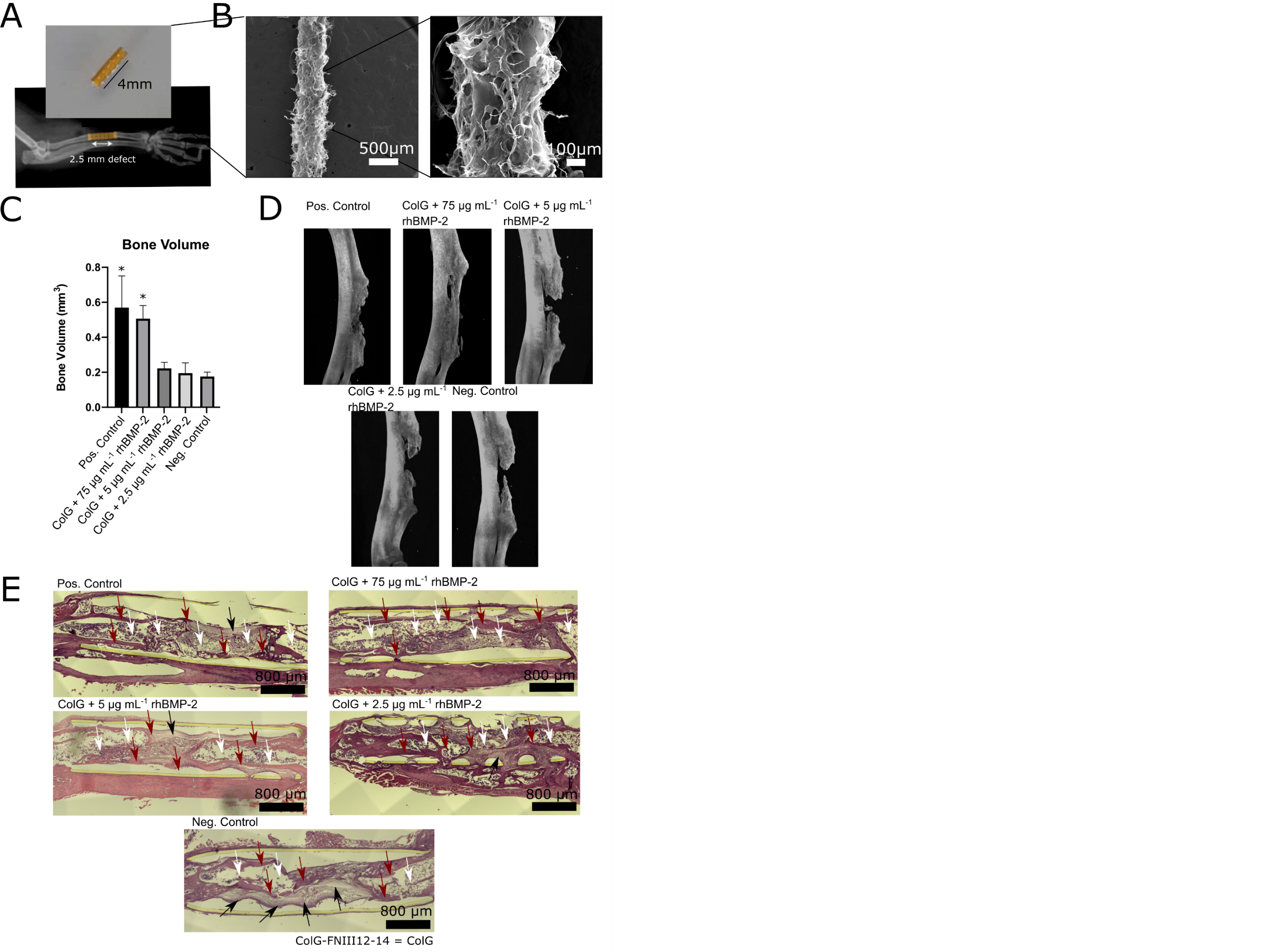


Supplementary Figure 8: Larger images of Figure 5E showing haematoxylin and eosin staining. Black arrows point to fibrotic tissue, white arrows point to the formation of a bone marrow cavity/cancellous bone, while maroon arrows point to new cortical bone. Pos. Control (75μg mL­_­_^-1^ rhBMP-2), neg. Control (plain sponge), Scale bar (800μm)
